# Modeling air-to-air communication networks in the North Atlantic region

**DOI:** 10.1007/s13272-023-00656-z

**Published:** 2023-05-02

**Authors:** Tobias Marks, Alexander Hillebrecht, Florian Linke

**Affiliations:** Institute of Air Transport, German Aerospace Center, Blohmstr. 20, 21079 Hamburg, Germany

**Keywords:** Communication, Ad-hoc networks, North Atlantic, Traffic simulation

## Abstract

The North Atlantic is one of the world’s airspaces accommodating a very high aircraft density while at the same time no radio coverage or radar surveillance is available. Beside satellite communication, one approach to enable data communication between aircraft and ground in the North Atlantic region is to establish ad-hoc  networks build up by direct data links between the aircraft that are acting as communication nodes. In this paper we, therefore, present a modeling approach to model air traffic and ad-hoc networks in the North Atlantic region using up-to-date flight plans and trajectory modeling techniques and to assess the connectivity provided by such networks. Assuming an applicable set of ground stations that provide data transfer to and from this airborne network, we assess the connectivity by time-series analysis and in total for a set of different fractions of all aircraft assumed to be equipped with the necessary systems as well as for a variation of the air-to-air communication range. In addition, we present average link durations, average amounts of hops to reach ground and numbers of connected aircraft for the different scenarios and identify general relations between the different factors and metrics. We will show, that communication range and equipage fraction significantly influence the connectivity of such networks.

## Introduction

The North Atlantic (NAT) is one of the most frequented airspaces in the world and, therefore, it is necessary not only to handle the current air traffic load but also to increase the capacity of the airspace to accommodate future air traffic growth [1]. As there is only very limited radar coverage in the border areas of the North Atlantic available, aircraft are widely separated in a timely manner in order to maintain safety resulting in a limited airspace capacity. Although the use of ADS-B over satellite already recently led to a reduction of separation minima [2] and might render the OTS obsolete in the future [3], the need for fallback solutions in case of a GNSS outage still persists.

Another drawback in the North Atlantic airspace results from the fact that air traffic control (ATC) communications relies on voice and controller-pilot data link communications (CPDLC) that is only possible via satellite (SATCOM) or HF/VHF [4]. Although SATCOM is available in the NAT region, this usually means higher monetary cost as it is often operated by private companies [5] as well as increased latencies [6, 7]. Therefore, it is not perfectly suited for many applications such as ATC, aeronautical operational control (AOC), airline administrative control (AAC), aeronautical passenger communication (APC) and others (see e.g. [8]). Although government-owned satellite communication infrastructure might be established in the future [9], current satellite systems coverage additionally might not be sufficient in remote areas. In addition, the upcoming mega constellations (e.g. Starlink or Oneweb) pose incalculable risks to low earth orbit (LEO) in terms of space debris [10] and rise the potential for future international conflicts extending into space [11] thus questioning the future reliability and availability of such systems.

Another way to establish communication in remote areas is to set up ad-hoc communication networks between aircraft while airborne using direct air-to-air (A2A) data links. Air-to-ground (A2G) communication is then rendered possible if some aircraft within this airborne network are connected to ground stations and are acting as gateways to transfer data between the airborne network and ground.

Beside providing direct A2A and A2G communication capabilities or constituting a backup for legacy communication systems, such a network can provide for future applications, such as wake energy retrieval, self-separation or online flightdata recording.

However, the operational usability of such an ad-hoc communication network strongly depends on the amount of aircraft within the airspace that are equipped with the necessary communications systems as well as on the geographic locations of the aircraft acting as network nodes. In addition, physical properties of the data link such as communication range strongly influence the topology of the created network and the availability of connections.

As also the ground communication infrastructure in continental areas is reaching its limits, in expectation of future air traffic growth, the L-band digital aeronautical communications system (LDACS) A/G data link represents the new future standard for data communication in continental airspaces (see e.g. [12, 13]) and is currently in the ICAO standardization process. LDACS A/G is specified in [14] and will render possible much higher data rates than traditional VHF data links. The development of a similar data link for A2A communications in the L-band was, therefore, under examination in the IntAirNet (Inter Aircraft Network) project that was funded by the German Ministry of Economic Affairs and Energy (BMWi) under the National Aeronautical Research Program (LuFo) V-3.

In order to derive requirements for such an A2A data link based on the LDACS technology, a detailed knowledge of the expected data traffic in terms of volume, packet sizes and frequency is essential. One part of the IntAirNet project, therefore, dealt with the development of a simulation environment being able to assess aircraft connectivity and data traffic on a global level. Beside the underlying flight movements of a scenario, two major parameters strongly influence the network topology and hence the connectivity and throughput: on the one hand the number of aircraft equipped with the new communication systems, on the other hand the range of the radio link under consideration. As a consequence, in our work we focus on this particular trade-off.

It needs to be mentioned, that although we focus in our work on the NAT region, there are other highly frequented airspaces existing where ad-hoc communication networks among aircraft might be feasible and that can be worthwhile to be analyzed in the future.

## Related work

*General Ad-hoc networks:* The concept of an ad-hoc network between flying aircraft was first envisioned by NASA for the Small Aircraft Transportation Systems (SATS) (see [15]) to provide A2A, A2G and ground-to-ground (G2G) communications to support different applications such as ATC, AOC and AAC services. The focus of their work lay on the network architecture and protocols. The idea was then adopted for commercial aircraft (see e.g. [16–19] or [20]) to provide a high bandwidth data link and was termed "Airborne Internet" or "Internet-Above-the-Clouds". While the underlying principle remains the same such a network can generally be referred to as an aeronautical ad-hoc network (AANET). A similar approach can be used for networks of unmanned aerial systems (UAS) (flying ad-hoc networks; FANETs) enabling a communication between the UAS without a ground infrastructure (see e.g. [21]), for vehicles (vehicular ad-hoc networks; VANETs) or in general for mobile nodes (mobile ad-hoc networks; MANETs). A valuable overview of AANET including the differentiation between the different types of ad-hoc networks is given by Zhang et al. [6] who are pointing out the challenges of designing AANETs.

*Applications:* Fundamental and dynamic changes of communication demand pose a severe challenge for typical commercial aviation innovation cycles. In the context of the expanding use of online services, previous works were, therefore, mainly targeting the high data traffic demand of passengers (APC) (see e.g. [8, 22, 23]). Other applications are described by Zhang et al. [6] who distinguish between fundamental applications (flight data delivery, air traffic control and tracking of aircraft) and enhanced applications (formation flight, free flight, in-flight entertainment). A distinct analysis of communication using AANETs for other applications than APC is not known to the autors.

*Mobility model and ground stations:* Among others Medina [8] adopted the AANET concept for the NAT region and was mainly focusing on feasibility and routing protocols using great circles for traffic modeling while assuming all aircraft flying at the same altitude. Here, only a very reduced set of 6 internet gateways provide ground connectivity in his simulations. Vey et al. [24] assessed connectivity and throughput based on real traffic traces over France and the North Atlantic. They distinguish between a continental and oceanic scenario accordingly. Zhang et al. assume three mobility scenarios namely flight over an airport or near an airport, flight over populated areas and flight over unpopulated areas.

*Communication range and coverage:* The strong dependency of the communication performance on equipage fraction (node density) as well as on communication range is obvious (see e.g. [6, 8]). Communication range in turn is mainly affected by the transmission power, the propagation of the radio signals through the atmosphere as well as on the radio horizon. The latter of which is determined by earth curvature and flying altitude.

As communication range and equipage fraction are limiting factors in ad-hoc communication networks, it is essential to understand how connectivity relates to changes in both parameters. Vieira et al. [25] analytically assessed link probability, node degree and network coverage. They conclude, that communication range is more significant than the number of nodes constituting the network. Medina et al. assessed in [18] several A2A communication ranges (100 nmi, 200 nmi, 300 nmi) in terms of connectivity assuming an equipage fraction of 1. In [8] Medina used in his study a communication range based on line-of-sight (LOS) distance while assuming an equipage fraction of 0.5. Vey et al. [24] assessed communication ranges of 100 km, 200 km and 400 km with a constant equipage fraction. They come to the conclusion, that for the oceanic scenario a communication range of 350 km is needed in order to provide a mean connectivity of more than 90 % for all aircraft during the day.

*Our contribution*: Despite the fact, that a lot of work concerning AANETS has been done in the past, a systematic analysis of the dependency of connectivity on the factors mentioned above that is based on actual flight plan data in combination with an advanced trajectory modeling process as presented in this paper is not known to the authors. Therefore, in this work we assess various connectivity metrics including number of hops, number of available connections and link duration for a systematic variation of equipage fraction and communication range. Hence, we provide a first decision support on what communication range needs to be realized in order to achieve a desired level of connectivity. Furthermore, our findings give a first hint on how AANET connectivity might develop while gradually deploying the new system in the future.

## Methodology

In our approach we model air traffic and the building of ad-hoc communication networks on the NAT in order to systematically analyze the influence of the A2A communication range as well as of the equipage fraction of aircraft on the attainable A2G and A2A connectivity as well as on link duration, number of hops and the number of available connections. In this section we will present the single aspects of our methodology in detail.

### General approach

The simulation environment, that was developed in the IntAirNet project and used to perform our studies, is called KOSMO and basically consists of two main modules one of which, the connectivity simulator (CSI), will be presented in this work. The basic approach for the second main module, the data traffic generator (DTG), is presented in [26].

**Fig. 1:**
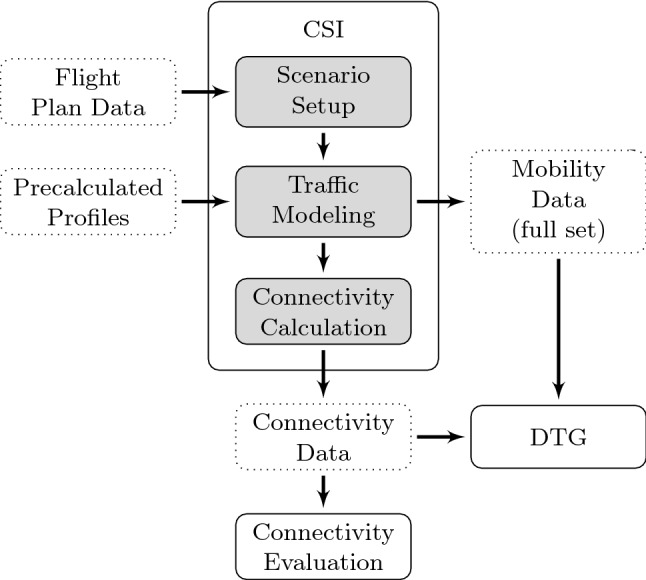
General approach as followed within this work

The general approach followed in our work is presented in Fig. 1. Following this approach, in a first step an air traffic scenario is created by the CSI containing relevant flights within the time frame under evaluation based on flight plan data. This scenario basically consists of a set of ground tracks with associated aircraft types, the positions of groundstations (see Sect. 3.3.4) and a definition of the simulation area under evaluation (see Sect. 3.3.3). Air traffic is then modeled using this data by mapping precalculated flight profiles to the intended ground tracks (see Sect. 3.5). The full set of mobility data created in this way is then on the one hand provided to the DTG to derive specific data traffic demand for all flights and on the other hand it is used to perform the connectivity calculation creating various sets of connectivity data depending on the boundary conditions used during the calculations (e.g. communication range and equipage fraction). The connectivity data is then passed to both the DTG and to a detailed connectivity evaluation process (see Sect. 3.6). The single steps of the CSI will be described in more detail in the individual sections below.

### Definitions and general assumptions

In this section some basic definitions are presented as they are used throughout this work. Table 1 gives an overview of the general definitions.

**Table 1:** General Definitions

Airborne station (AS)	An aircraft acting as a flying node
Ground station (GS)	A radio station that is assumed to be fixed on the ground
A2G connection	A connection between an AS and a GS is given, if the AS can communicate directly or indirectly with a GS. A *direct A2G connection* is given if the AS is situated within the assumed communication radius of a GS (one hop). An *indirect A2G connection* is given if the AS is connected via at least one hop to another AS that is located within direct A2G connection with a GS
A2A connection	A connection between two AS is given, if the distance between both AS is less than the assumed communication range of the A2A data link radio and the AS are within visual range of each other as defined in Sect. 3.2
Link	A link is understood to be a radio connection between two AS or an AS and a GS that is persistent over a certain period of time
Hop	We define a hop being one edge of the path on the network graph that is used in order to establish an A2G connection
Setting	We define a setting as a combination of parameters as presented in Table 2 that are specifying a scenario
Scenario	We define a scenario as being a particular implementation of a setting including a randomized selection of equipped aircraft

*Earth model:* Throughout our work we approximate the surface of the earth by the WGS84 earth ellipsoid. Only the estimation of the maximum line-of-sight (LOS) distance is based on a spherical earth model for simplification.

*Radio propagation model*: In our model we assume an omni-directional propagation of the radio signals to determine connectivity between two AS or between AS and GS. Hereby the communication range is limited by two main factors. This is on the one hand the radio horizon, which is dependent on the altitudes of the two stations in question, and on the other hand the performance of the radio transmitters and receivers, that is mainly affected by available power, antenna design and the propagation environment (in our case the atmosphere).

It can be expected that the latter represents the limiting factor for communication range in both A2A and A2G cases, as depending on the chosen communication technology atmospheric effects can be significant, while the available transmission power is limited. As this paper uses the LDACS technology as a baseline, the technical communication range for A2G communication can not exceed the LDACS design range of 200 nmi as in the LDACS specification [13]. However, in our study the maximum communication range of the A2A link ($r_a$) is variable (see Sect. 3.4) in order to identify technical requirements for the A2A system (see Sect. 4.3).

Concerning the radio horizon it can be expected, that within ORP airspaces aircraft usually fly at high altitudes resulting in radio horizons extending far beyond the design range. However, radio horizon limitations might occur during descent or climb phases especially while evaluationg A2G connections. Therefore, in our model radio horizon effects are covered. Here, although we use the WGS84 ellipsoid to approximate earth, the maximum LOS distance $r_{\text {LOS}}$ between two AS flying at altitudes $a_{1}$ and $a_{2}$ is approximated by equation 1 assuming a spherical earth (see e.g. [8]) as it can be expected, that the errors due to this simplification are neglectable, while at the same time calculation time and complexity of the model are significantly reduced.

1 $$\begin{aligned} r_{\text {LOS}} = \sqrt{a_{1}^{2} + 2 R_{e} a_{1}^{2}} + \sqrt{a_{2}^{2} + 2 R_{e} a_{2}^{2}}. \end{aligned}$$

To generate the network topology, the communication ranges, both for A2G ($r_{g}$) and A2A ($r_{a}$) communication, are assumed to be uniformly distributed in all directions representing a unit sphere model using the Euclidean distance between two nodes as distance metric. Hence, if the Euclidean distance between the two AS is smaller than $r_{\text {LOS}}$ and below $r_{a}$ an A2A connection is assumed to be established. The equivalent definition is applied for A2G connections.

### Scenario setup

In this section the setup of the base scenario is described that was used for all studies presented in this work.

#### Traffic data

For the modeling of air traffic flight plan data from first of August 2019 was taken from the Sabre Market Intelligence database [27]. The data was then filtered to only contain flights between Europe and North America. In order to reduce the amount of data, an additional two staged filtering was applied, reducing the flights to long range aircraft in the first step and limiting it to flights with ranges above 1000 km in a second step.

#### Flight direction

Air traffic on the NAT is separated geographically by the organized track system (OTS) in order to account for changing wind and traffic conditions. As in our simulation we use geodesics as flight tracks (see Sect. 3.5) it is not feasible to include both flight directions into one common simulation as this separation is not reproduced and hence collisions of aircraft might occur.

**Fig. 2:**
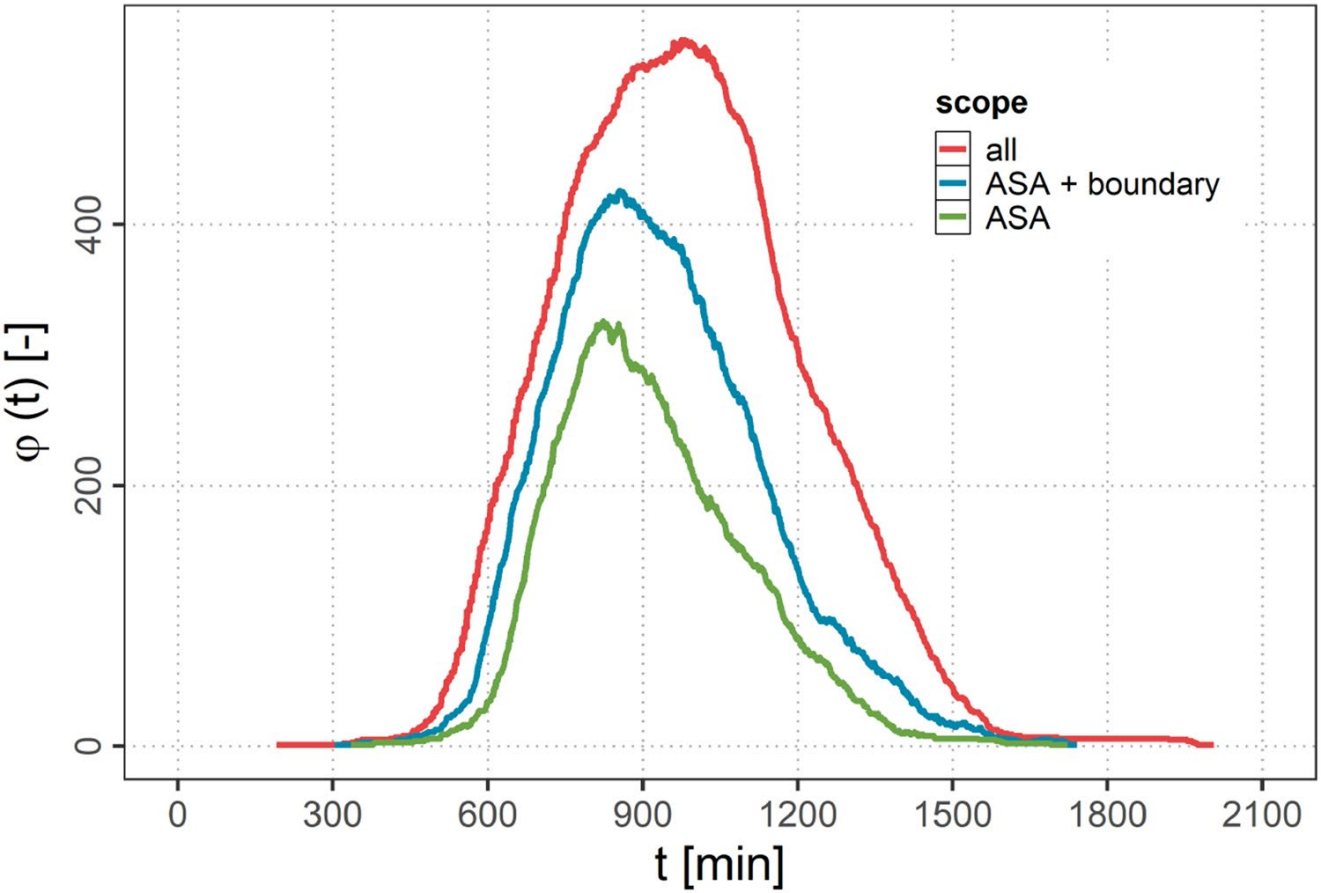
Amount of aircraft over time in westbound flight direction for the selected flights (red curve), the flights in the ASA and boundary region (blue curve) and flights in the ASA only (green curve)

However, as air traffic going over the NAT mainly consists of two waves during the day (one westbound traffic flow and one eastbound traffic flow) that only partially overlap in a timely manner in the off-peak hours, it is feasible to separate these two waves and assess them separately. In our studies we, therefore, focus exemplary on the westbound traffic flow and exclude all eastbound traffic resulting in 665 flights in total.

Figure 2 shows the resulting amount of aircraft $\varphi (t)$ of the westbound traffic flow over the simulation time *t* as defined in Sect. 3.6.1 for the whole scenario (red curve).

#### Applicable simulation area

As it can be expected that data communication is not provided primarily in oceanic, remote and polar (ORP) airspaces, in our model it is assumed, that the applicable simulation area (ASA) with no data link availability is located in the oceanic control areas (OCA) on the North Atlantic as listed below.

- Gander (CZQX)
- Shanwick (EGGX)
- Bodo (ENOB)
- Reykjavik (BIRD)
- New York (KZWY)
- Santa Maria (LPPO)

Additionally to the constraints given by these OCA boundaries, in our model the ASA is limited southwards by the 39th parallel. All evaluations presented in this paper, therefore, only address flights or parts of flights within the ASA. If a flight leaves the ASA it is not longer subject to evaluation.

However, a flight leaving the ASA still holds the potential to establish an air connection across the ASA border and can act as a gateway for other aircraft to establish an indirect ground connection. In order to allow these connections to be considered in our study, a buffer range around the ASA is defined. The offset of this buffer range $r_{b}$ was selected to be 420 km according to the projected variation of the A2A communication range $r_{a}$ (see Sect. 3.2) ensuring no such cross boundary connections are missed, while at the same time reducing the dataset as much as possible in order to reduce computation time.

**Fig. 3:**
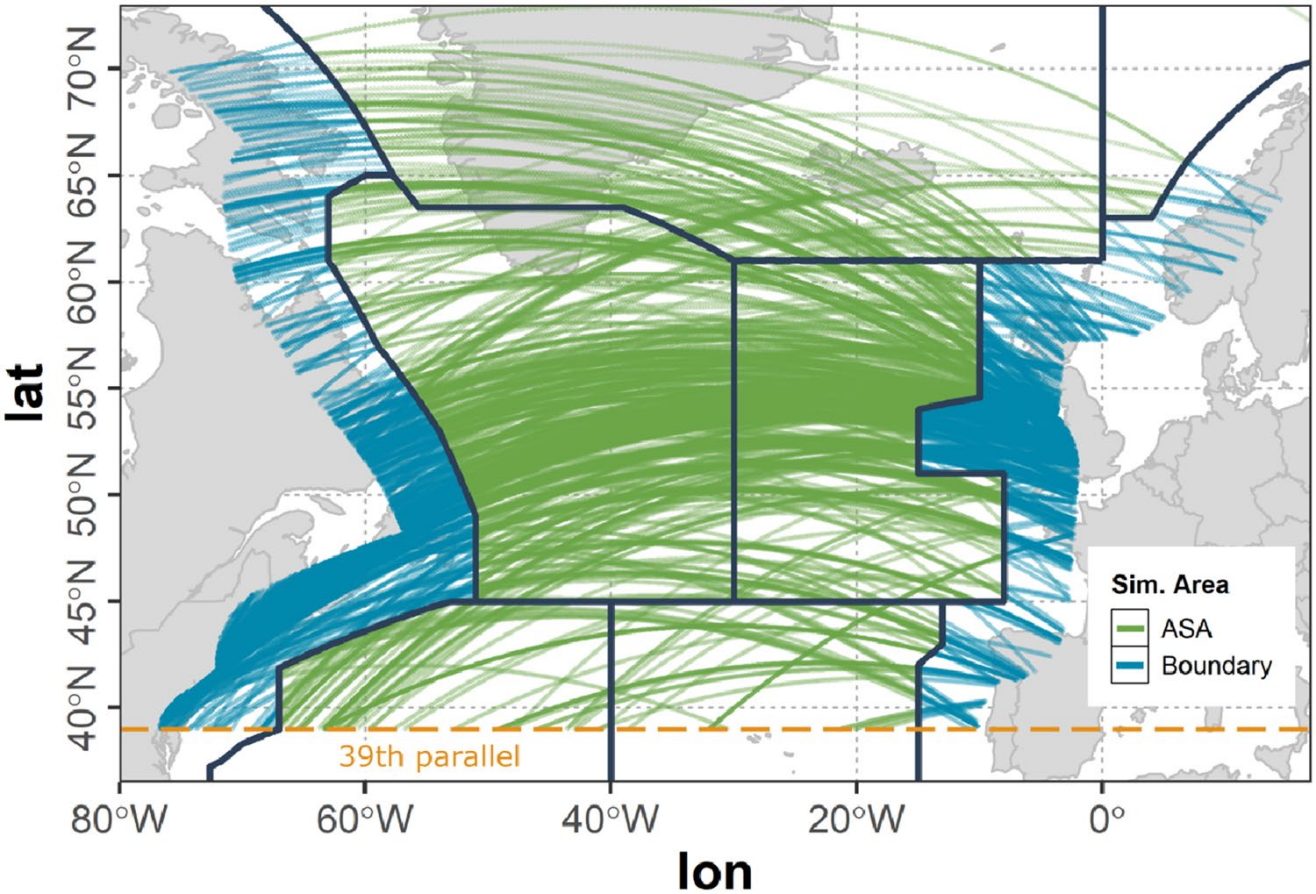
Flights of the westbound traffic scenario within the ASA and the boundary region

Figure 3 shows the ASA used in the studies presented within this paper as described above. The parts of flights of the full scenario within the ASA are colored green whereas all parts of flights within the ASA boundary are colored blue. Parts of flights outside the ASA and the boundary are not considered.

Figure 2 shows the amount of aircraft over time based on the mobility data used in our study (see Sect. 3.3.1) of the full tracks of the selected flights (red curve), the flights in the ASA and boundary region (blue curve) as well as of the flights in the ASA only (green curve). It can be observed, that a strong reduction of data is achieved (red curve versus blue curve) that is as a consequence drastically speeding up the connectivity calculations during the course of the study.

#### Ground stations

**Fig. 4:**
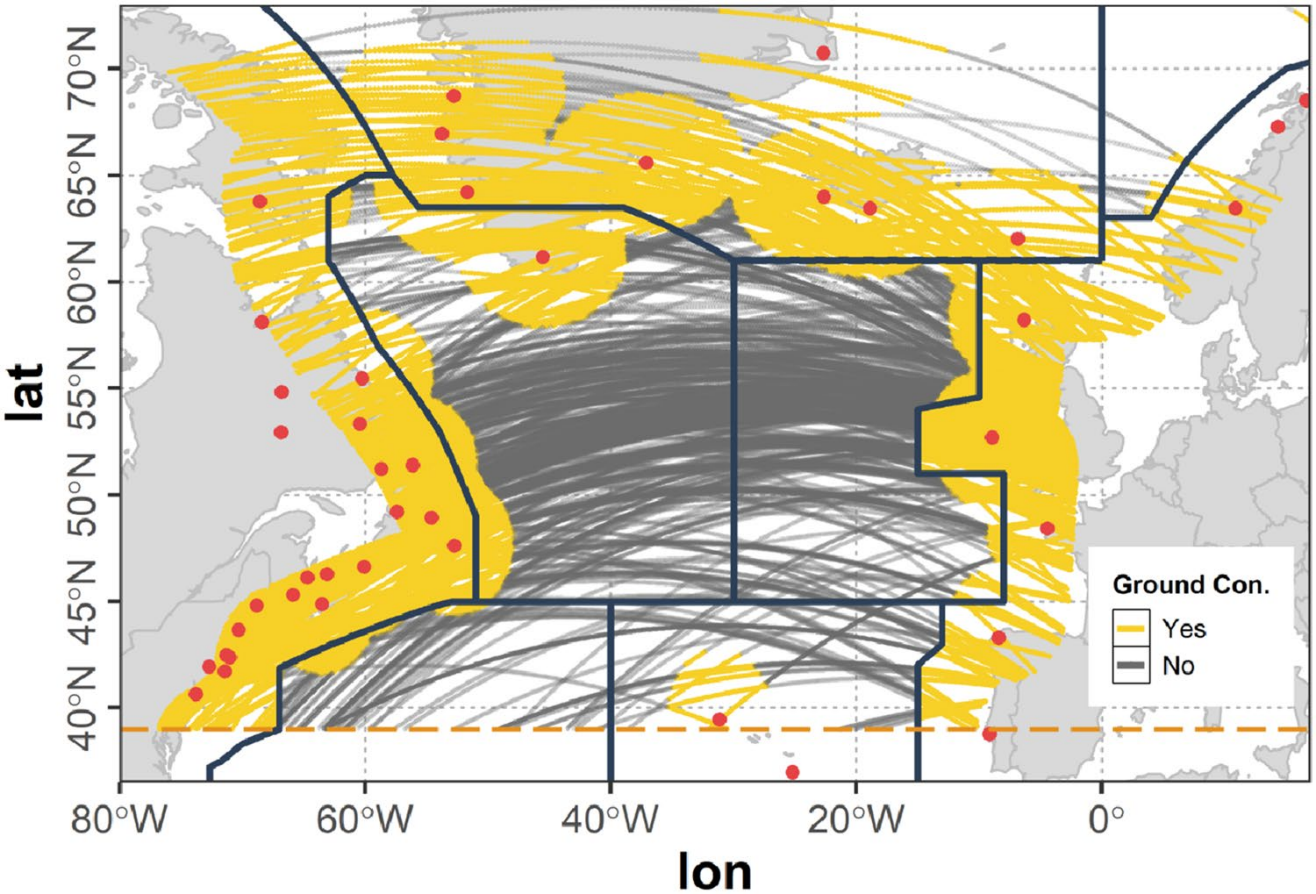
Flights of the westbound traffic scenario within ground station coverage assuming $r_{g} = 370\,\text{km}$; geographic locations of ground stations (red)

In our simulation setup 47 ground stations were assumed to act as transitional nodes for the airborne network (see Fig. 4). The geographic locations and altitudes of the ground stations (red) were assumed to be identical with present VHF ground stations from ARINC and SITA. The maximum radio range for A2G communication $r_{g}$ was assumed to be 370 km (see Sect. 3.2) according to the LDACS A/G design range which is 200 nmi (see [14]) and in accordance with [18]. To model the A2G connectivity a unit sphere model was used (see Sect. 3.2). As this simple modeling approach cannot cover the real situation, additionally all parts of flights outside the ASA that are situated within the ASA boundary are considered to be in ground coverage. In Fig. 4 all parts of flights within direct ground connection are colored yellow whereas parts of flights without direct ground connection are colored gray.

The resulting coverage map shown in Fig. 4 roughly corresponds to the coverage map provided by ICAO in [4]. However, as in this document by ICAO it is stated, that the maps are outdated, further information on the ground coverage is needed in the future to more accurately asses the ground station coverage. In our flexible simulation environment additional stations incorporating even individual communication ranges can easily be added as soon as the data is available.

### Variations

It can be assumed, that not all aircraft are equipped with the necessary communications systems straight after the technology is introduced. Instead the fraction of equipped aircraft will begin with small numbers and increase over time. One important question is, therefore, how large the equipage fraction needs to be in order to enable a functioning communication network providing an acceptable connectivity.

For this reason, different equipage fractions $e_\textrm{f}$ of the original data were selected for investigation. In this selection process a certain percentage of flights is randomly removed from the full flight plan. In order to get a decent understanding of the influence of $e_\textrm{f}$ on the different connectivity metrics, we used a sequential variation of 10 % in our studies. To account for uncertainties resulting from the random selection process we additionally calculated a set of $n_{ smp }=10$ random samples for each fraction level using the Mersenne-Twister as pseudo-random number generator.

Beside the influence of the equipage fraction $e_\textrm{f}$ it is important to get an understanding on how the maximum A2A communication range $r_{a}$ influences connectivity between the equipped aircraft. As a consequence, this parameter was also subject to variation in our studies. Here, in order to get a decent understanding of the influence of $r_{a}$, a sequential variation from 0 km to 420 km was applied employing steps of 15 km.

Table 2 presents a summary of the scope of variations as used in the study.

**Table 2:** Parameter variations

Symbol	Variation	Unit
$r_{g}$	370	km
$e_\textrm{f}$	0: 0.1: 1	–
$r_{a}$	0: 15: 420	km

### Traffic modeling

**Fig. 5:**
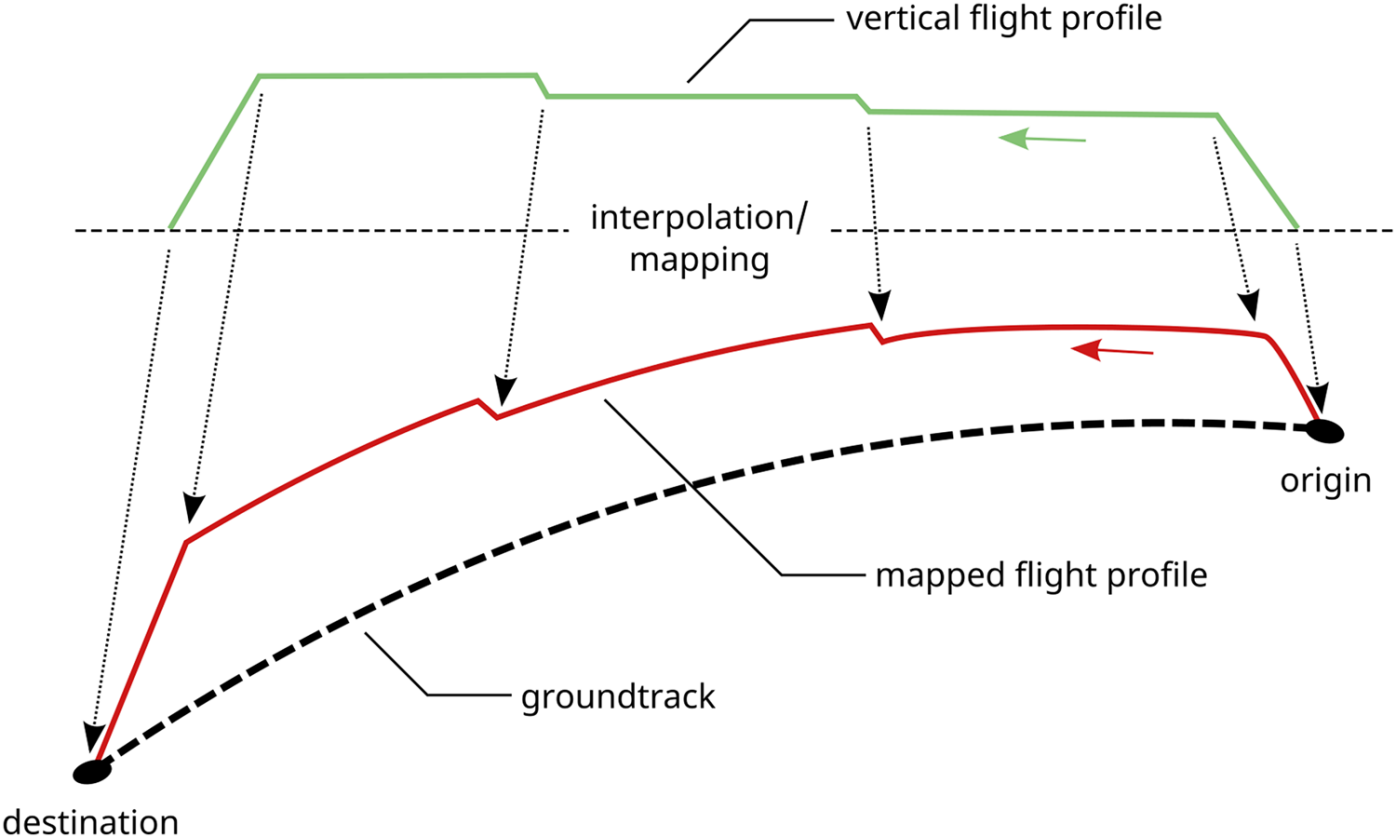
Schematic process of mapping of a trajectory to a geodesic

In our modeling of the individual flights we use precalculated trajectories generated by the trajectory calculation module (TCM) [28], a software tool developed by the German Aerospace Center (DLR). The TCM is based on the EUROCONTROL base of aircraft flight performance models and data (BADA) [29] in version 4. The precalculated trajectories are selected by aircraft type and ground distance and are subsequently projected on the geodesics generated for each flight contained in the filtered flight plan data as shown schematically in Fig. 5. To select the appropriate trajectory from the database, a passenger load factor of 0.85 was assumed, representing a mean value for the NAT area [30] before the COVID-19 pandemic.

All trajectories that are created by this process are subsequently synchronized by interpolation in order to assure synchronous timestamps throughout the simulation. In all our simulations we use a chronological discretization of 60 s.

As the precalculated trajectories contain detailed vertical flight profiles and, therefore, include top of climb, top of descent as well as step climbs, our traffic modeling approach enables a detailed geographic triggering of data traffic connected to these specific events that are handed over to the DTG as described in Sect. 3.1. Additionally this approach enables LOS assessment by correctly determining altitudes and viewing angles between the AS.

### Connectivity assessment

In this section methods are being presented on how to assess connectivity in our simulation. Here, it is basically differentiated between A2G and A2A connectivity. As stated in Sect. 3.3.3 the connectivity assessment is only performed for the parts of flights located within the ASA.

#### Amount of aircraft

The set of all aircraft that are situated within the ASA at timestamp *t* is given by *S*(*t*). The amount of aircraft within the ASA at timestamp *t* is given by the cardinality of *S* and is denoted by $\varphi (t)$.

2 $$\begin{aligned} \varphi (t) = \vert S(t) \vert . \end{aligned}$$

#### Air-to-ground connectivity

The set of aircraft within the ASA at timestamp *t* having a ground connection is given by $S_{g}(t)$ and the corresponding amount of aircraft can be denoted as $\varphi _{g}(t)$ accordingly. The ground connectivity $C_{g}(t)$ at time *t* can then be defined by the fraction of the amount of connected aircraft to the amount of all aircraft.

3 $$\begin{aligned} C_{g}(t)=\frac{\varphi _{g}(t)}{\varphi (t)}. \end{aligned}$$

In order to distinguish between direct and indirect ground connectivity indices are used representing the number of hops necessary to reach ground. The number of hops necessary for an AS to reach the nearest GS is denoted as $n_{h}$. In our simulation the shortest path to connect to a GS is determined during the calculation process.

Following this naming schema, $C_{g1}(t)$ denotes the relative fraction of direct ground connections (one hop; $n_{h}=1$) at timestamp *t* whereas $C_{g>1}(t)$ denotes the relative fraction of indirect ground connections (more than one hop; $n_{h}>1$) at timestamp *t*.

As the simulation is discretized in 60 s steps the overall accumulated flight time expressed in minutes of the parts of flights flown in the ASA $\tau$ can be calculated by

4 $$\begin{aligned} \tau =\sum _{t}\varphi (t). \end{aligned}$$

Accordingly the accumulated flight time with ground connections $\Gamma$ can be defined as

5 $$\begin{aligned} \Gamma =\sum _{t}\varphi _{g}(t) \end{aligned}$$

The relative fraction $\gamma$ of the accumulated flight time with ground connection to the overall accumulated flight time $\tau$ is then given by

6 $$\begin{aligned} \gamma =\frac{\Gamma }{\tau }. \end{aligned}$$

In order to distinguish between direct and indirect ground connectivity for the scenario indices are used as already mentioned above. An important metric that will be used in the subsequent study is $\gamma _{0}$, describing the relative fraction of the accumulated flight time with no ground connection (no hop; $n_{h}=0$). $\gamma _{1}$ accordingly denotes the relative fraction of the accumulated flight time with direct ground connection (one hop; $n_{h}=1$) whereas $\gamma _{>1}$ denotes the relative fraction of the accumulated flight time with indirect ground connection (more than one hop; $n_{h}>1$). Other fractions are labeled accordingly.

As each hop in a multi-hop connection adds a delay to the communication, the more hops are needed to establish a ground connection the stronger the communication will be affected in terms of lag time when messages travel back and forth between sender and receiver. Therefore, another important figure to characterize the A2G connectivity of a scenario is the average number of hops needed to reach ground ($N_{h}$) as defined by the sum over the simulation time *t* and over the number of hops $n_{h}$ of all aircraft in ground connection $S_{g}(t)$ divided by the accumulated flight time in ground connection $\Gamma$.

7 $$\begin{aligned} N_{h}=\frac{\sum _{t}\sum _{i} n_{hi}(t)}{\Gamma } \quad \forall i \in S_{g}(t). \end{aligned}$$

The number of hops for each connection is determined in our study by calculating the shortest path between the AS and the next available GS. In reality other routes might be taken in order to establish a stable connection depending on the routing protocol in use (see e.g. [31] or [6]). However, this specific effect as well as lag time is not covered within our model.

#### Air-to-air connectivity

Similar to the definition of the ground connectivity, the amount of aircraft within the ASA at time *t* having an air connection is denoted by $\varphi _{a}(t)$. The air connectivity $C_{a}(t)$ can then be calculated at time *t* by

8 $$\begin{aligned} C_{a}(t)=\frac{\varphi _{a}(t)}{\varphi (t)}. \end{aligned}$$

In order to distinguish between the amount of connected aircraft indices are used. $C_{a1}(t)$ denotes the relative fraction of aircraft having exactly one air connection at timestamp *t* whereas $C_{a>10}(t)$ denotes the relative fraction of aircraft that have more than 10 air connections at timestamp *t*. Other indices can be interpreted accordingly. With *A* being the accumulated flight time with air connections according to

9 $$\begin{aligned} A=\sum _{t}\varphi _{a}(t), \end{aligned}$$

the relative fraction $\alpha$ of the accumulated flight time with air connections *A* in relation to the accumulated amount of flight time $\tau$ is given by

10 $$\begin{aligned} \alpha =\frac{A}{\tau }. \end{aligned}$$

In order to distinguish between the number of connected aircraft indices are used accordingly. An important metric that will be used in the subsequent study is $\alpha _{0}$ , describing the relative fraction of the accumulated flight time with no air connections ($n_{c}=0$). $\alpha _{1}$ denotes the relative fraction of the accumulated flight time with exactly one air connection ($n_{c}=1$) whereas $\alpha _{>1}$ denotes the relative fraction of the accumulated flight time with more than one air connection ($n_{c}>1$). Other fractions are labeled accordingly.

Another important figure to characterize the A2G connectivity of a scenario is the average number of connections ($N_{c}$) as defined by the sum over the simulation time *t* and over the number of connections $n_{c}$ of all aircraft with air connection $S_{a}(t)$ divided by the accumulated flight time with air connection *A*.

11 $$\begin{aligned} N_{c}=\frac{\sum _{t}\sum _{i} n_{ci}(t)}{A} \quad \forall i \in S_{a}(t) \end{aligned}$$

#### Link duration

An A2A link *l* is given, if two AS are situated within communication range for more than one coherent timestamp. The duration *d* of a link *l* is defined as the number of subsequent timestamps during which the link is stable without breaking up. The same definition applies for A2G links. The set of all links between AS *i* and another arbitrary AS is accordingly denoted as $l_{i}$ whereas the set of all links between any AS in the considered scenario is denoted as *L*. The average link duration for all links *L* in a scenario is then given by

12 $$\begin{aligned} D=\frac{\sum _{n} d}{\vert L \vert } \quad \forall n \in L. \end{aligned}$$

It needs to be noted, that links are applicable while at least one AS is located within the ASA as defined in Sect. 3.3.3. This means, that if one AS participating in a link leaves the ASA, the link is still considered stable until both AS are located outside of the ASA.

## Results

In this section exemplary results are being presented. Here, an analysis of three representative scenarios is followed by an assessment of the influence of the equipage fraction $e_{f}$ and maximum A2A communication range $r_{a}$ on the connectivity metrics.

### Scenario analysis

In this section an analysis will be performed for three representative scenarios. The corresponding scenario settings are presented in Table 3. While the flight direction and A2G communication range $r_{g}$ remain unchanged for all scenarios, the equipage fraction $e_\textrm{f}$ and the maximum A2A communication range $r_{a}$ are varied. The example scenarios were selected in order to get a first impression of the influence of both factors. Here, while an equipage fraction of 0.8 seems realistic if the system is already in a mature state, the fraction of 0.4 was selected to represent an intermediate step in the deployment process of the system. Additionally a maximum A2A communication range of 330 km seems realistic when referring to the LDACS specification, while a range of 150 km was selected to represent a strongly reduced range.

For all exemplary scenarios the selection of aircraft was performed in a random fashion as described in Sect. 3.4.

In the following a time-series analysis will be presented for all exemplary scenarios followed by an analysis of the distribution of necessary hops, available connections as well as link durations.

**Table 3:** Settings for example scenarios

Parameter	Setting A	Setting B	Setting C
Direction	West	West	West
$e_\textrm{f}$	0.8	0.8	0.4
$r_{a}$	330 km	150 km	330 km

#### Ground Connectivity

**Fig. 6:**
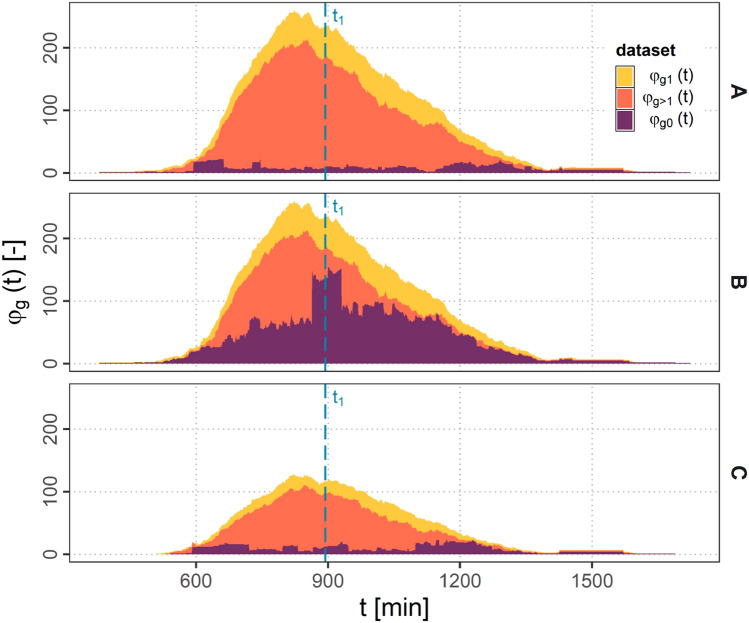
Stacked area plot of $\varphi _{g}(t)$ over simulation time *t* for scenarios A, B and C

In Fig. 6 the amount of aircraft with ground connection $\varphi _{g}$ over the simulation time *t* is presented as stacked area plots. As $e_\textrm{f}$ is equal in scenarios A and B the total amount of aircraft as well as the fraction of directly connected aircraft $\varphi _{g1}$ is identical for both scenarios. Scenario C shows less aircraft in total, as $e_\textrm{f}$ is considerably lower. Therefore, also the amount of directly connected aircraft $\varphi _{g1}$ is decreased significantly.

**Fig. 7:**
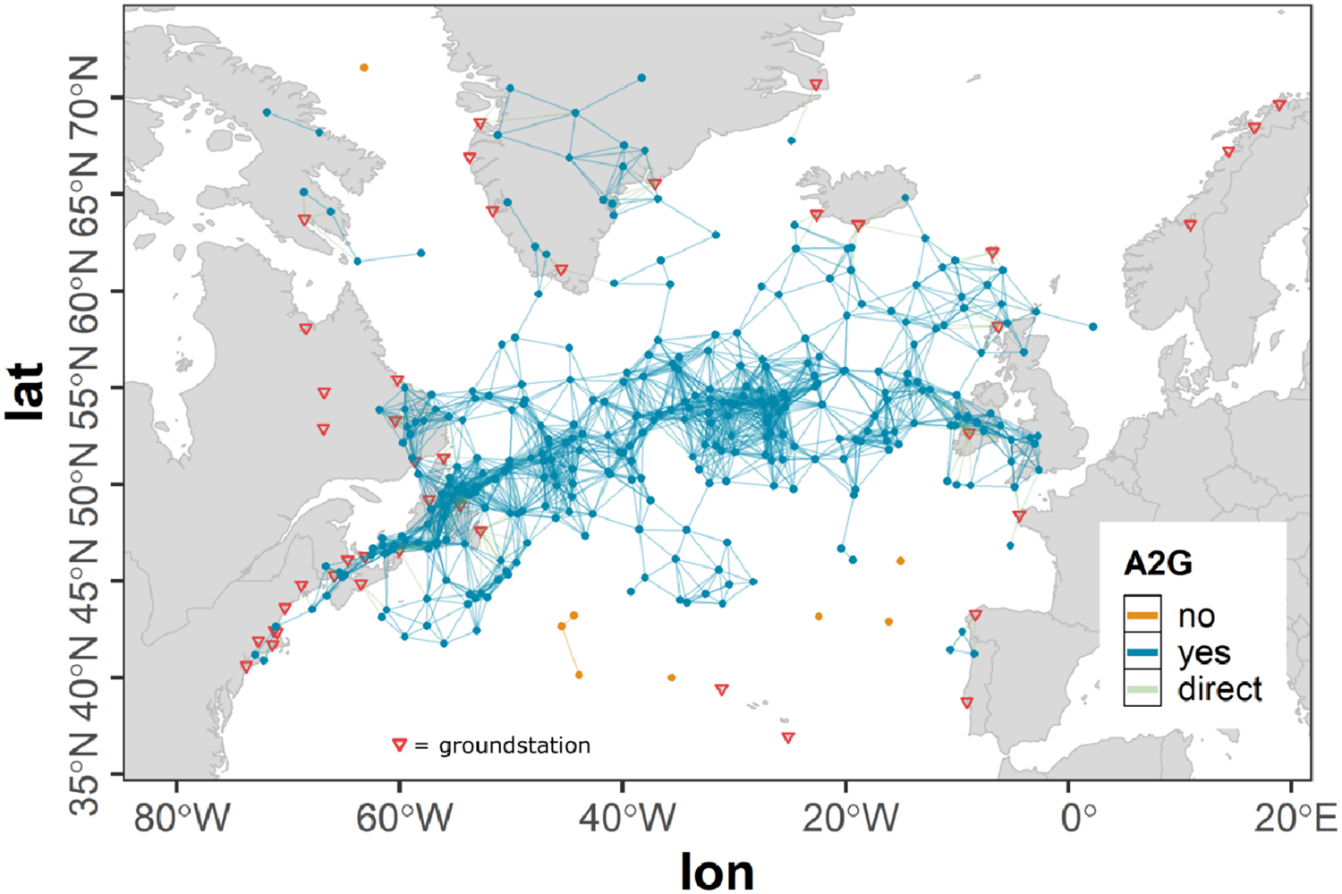
Connectivity for scenario A at timestamp $t_{1}$

**Fig. 8:**
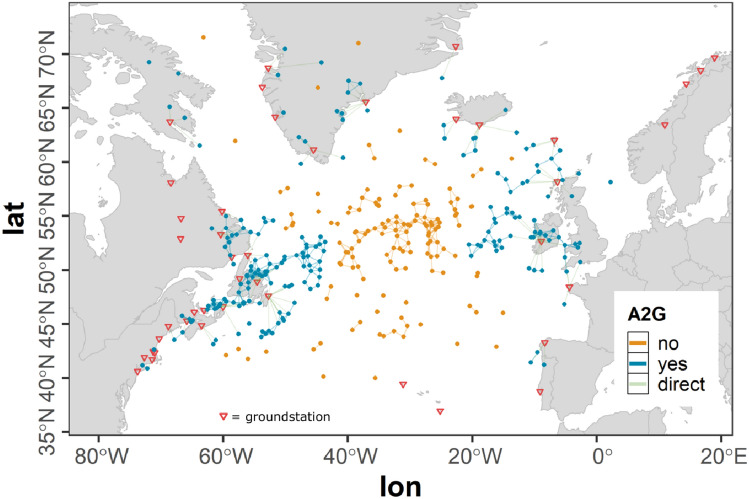
Connectivity for scenario B at timestamp $t_{1}$

**Fig. 9:**
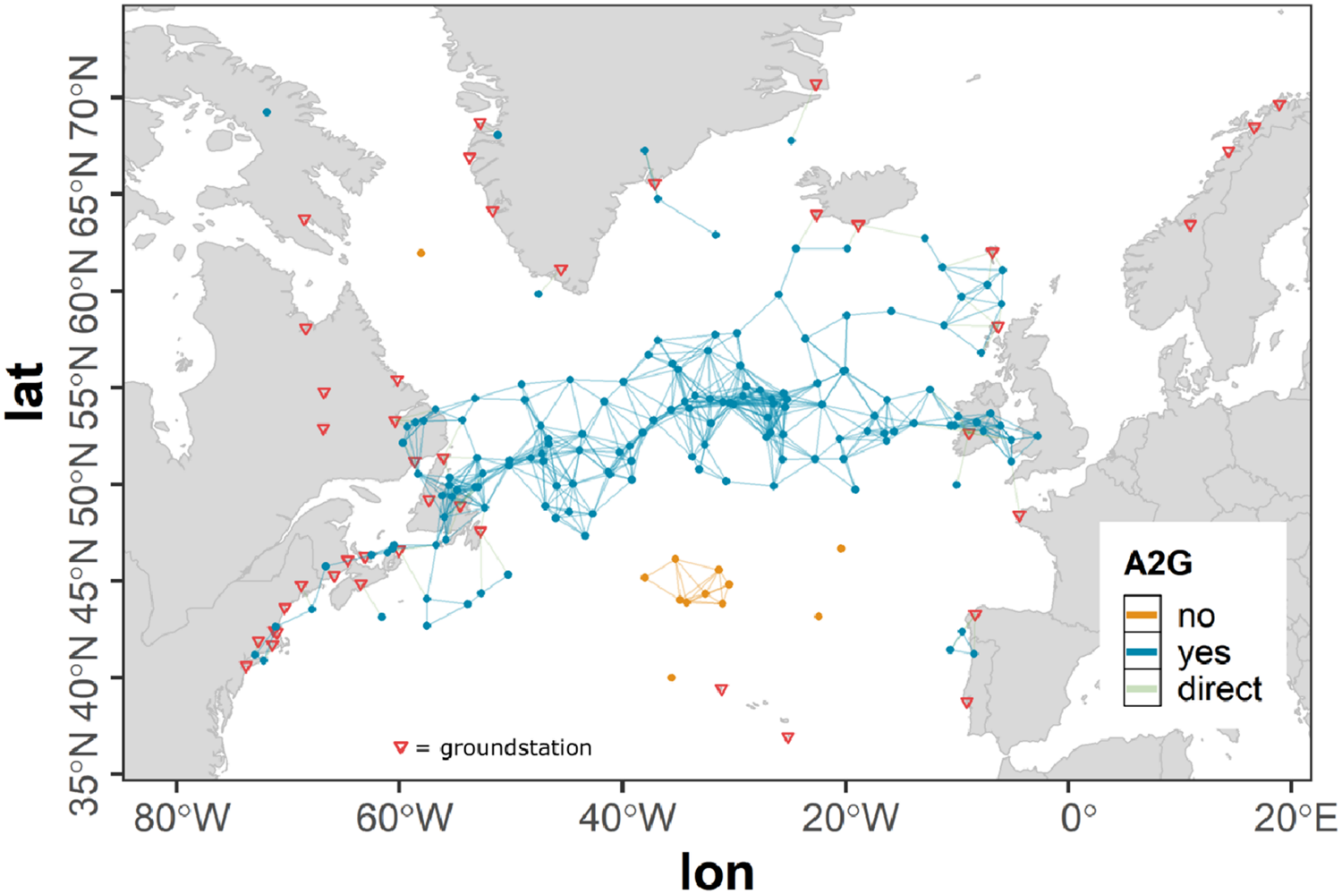
Connectivity for scenario C at timestamp $t_{1}$

However, there are more drastic changes concerning the indirectly connected aircraft $\varphi _{g>1}$ and aircraft without connection to the ground $\varphi _{g0}$. While $\varphi _{g0}$ is rather low in scenario A and C, scenario B shows a considerably higher amount of not connected aircraft. Furthermore, it can be observed, that in Scenario B a peak in the $\varphi _{g0}$ share indicates a sudden change in connectivity. To further investigate this, the traffic and connectivity situation on the ASA for a distinct timestamp $t_{1}$ within this peak for all three scenarios is presented in Figs. 7, 8 and 9.

It can be observed, that in scenario A and C a *connectivity bridge* forms over the NAT connecting Europe an North America. Only some individual airplanes or smaller clusters of airplanes are not connected to the ground.

In scenario B in contrast it can be observed, that a large cluster of interconnected aircraft is isolated without any ground connections. The cluster in this particular case was identified to be separated from ground after disconnecting on the European side of the NAT for a time period of about 66 min until it reconnected in North America again. This effect can be attributed to the reduced $r_{a}$. Beside the big isolated cluster also many small separated clusters are formed in this scenario due to the reduced $r_{a}$.

Scenario C shows the same timestamp with a reduced $e_{f}$ and the same $r_{a}$ as in scenario A. It can be observed, that although less aircraft than in scenario B are building up the network, due to the higher $r_{a}$ still a communication bridge is formed over the NAT connecting the two continents.

#### Air connectivity

**Fig. 10:**
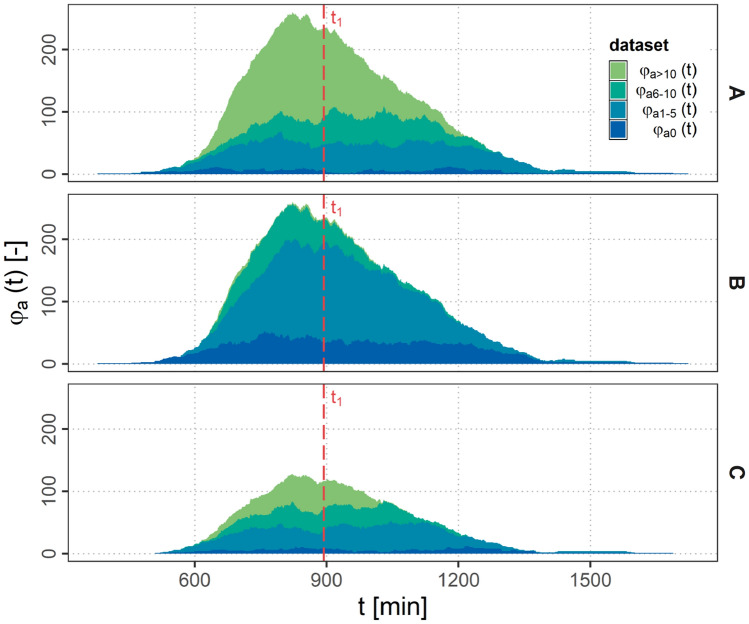
Stacked area plot of $\varphi _{a}(t)$ over simulation time *t* for scenarios A, B and C

In Fig. 10 the air connectivity $\varphi _{a}$ over the simulation time for different numbers of connected airplanes is presented as stacked area plots. It can be seen, that in scenario A and C almost all aircraft are connected to at least one other aircraft. As a result of the reduced $r_{a}$ in scenario B the share of not connected aircraft ($\varphi _{a0}$) is considerably higher than in scenario A and C. Also the amount of aircraft with more than 5 and up to 10 ($\varphi _{a6-10}$) is much less in scenario B. Aircraft with more than 10 ($\varphi _{a>10}$) connections are very rare.

Figure 10 shows that the number of connected airplanes is strongly depending on $r_{a}$ whereas $e_{f}$ more influences the amount of connected airplanes in total.

#### Hops for A2G connection

**Fig. 11:**
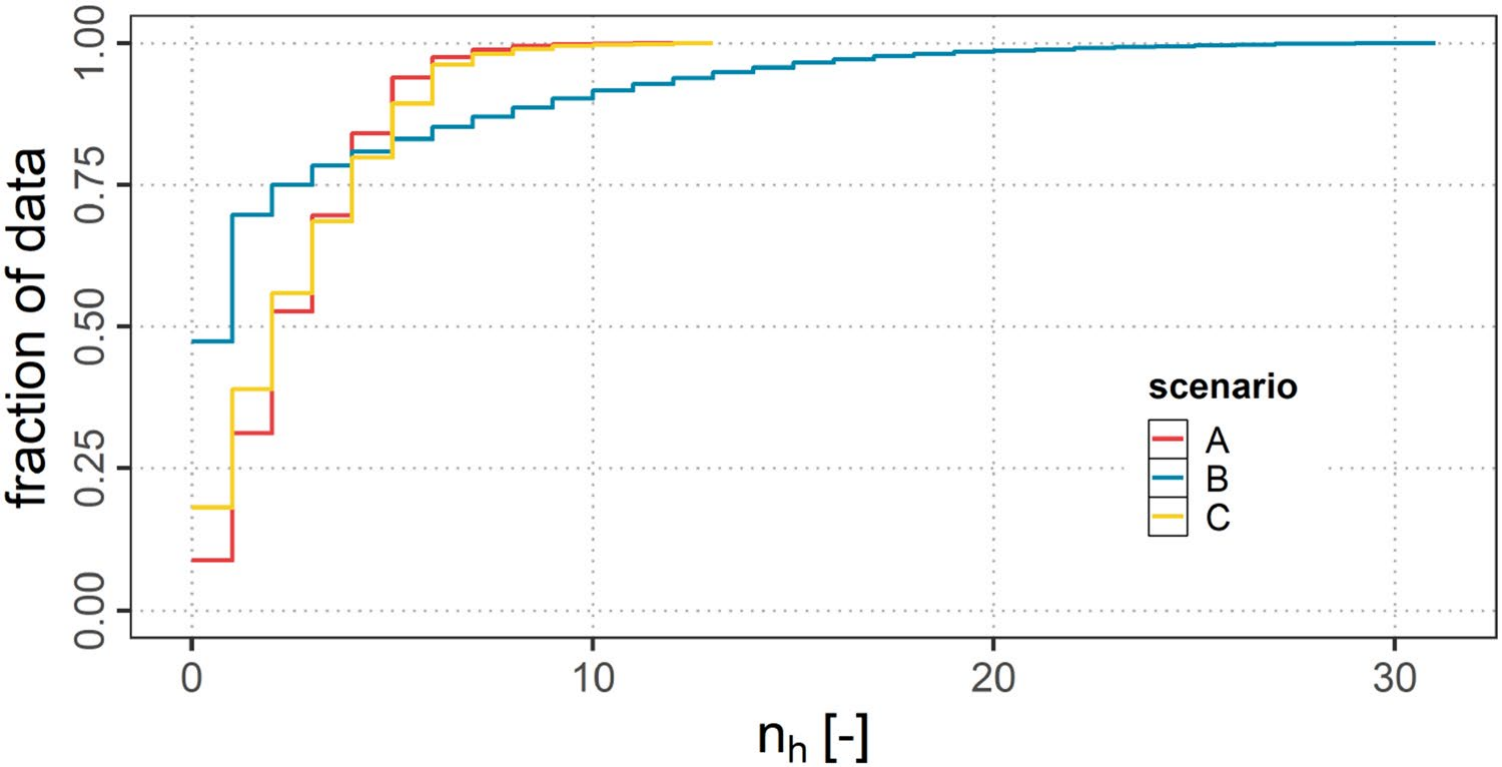
CDF of $n_{h}$ for scenarios A, B and C

Figure 11 shows a cumulative distribution function (CDF) of the number of hops $n_{h}$ necessary for an aircraft in order to reach ground for all three scenarios. It can be observed, that in scenario A and C only a small fraction of aircraft has no ground connection ($n_{h}=0$), whereas in scenario B almost 50 % of the flight time within the ASA aircraft are not connected. In contrast to A and C, scenario B additionally shows higher numbers of hops ($n_{h}>30$) whereas in scenarios A and C the maximum number of hops $n_{h}$ is 12 and 13. This indicates that if many nodes are in the network, ground connections still can be established even at smaller $r_{a}$ values, however, needing more hops to reach ground.

#### Available connections

**Fig. 12:**
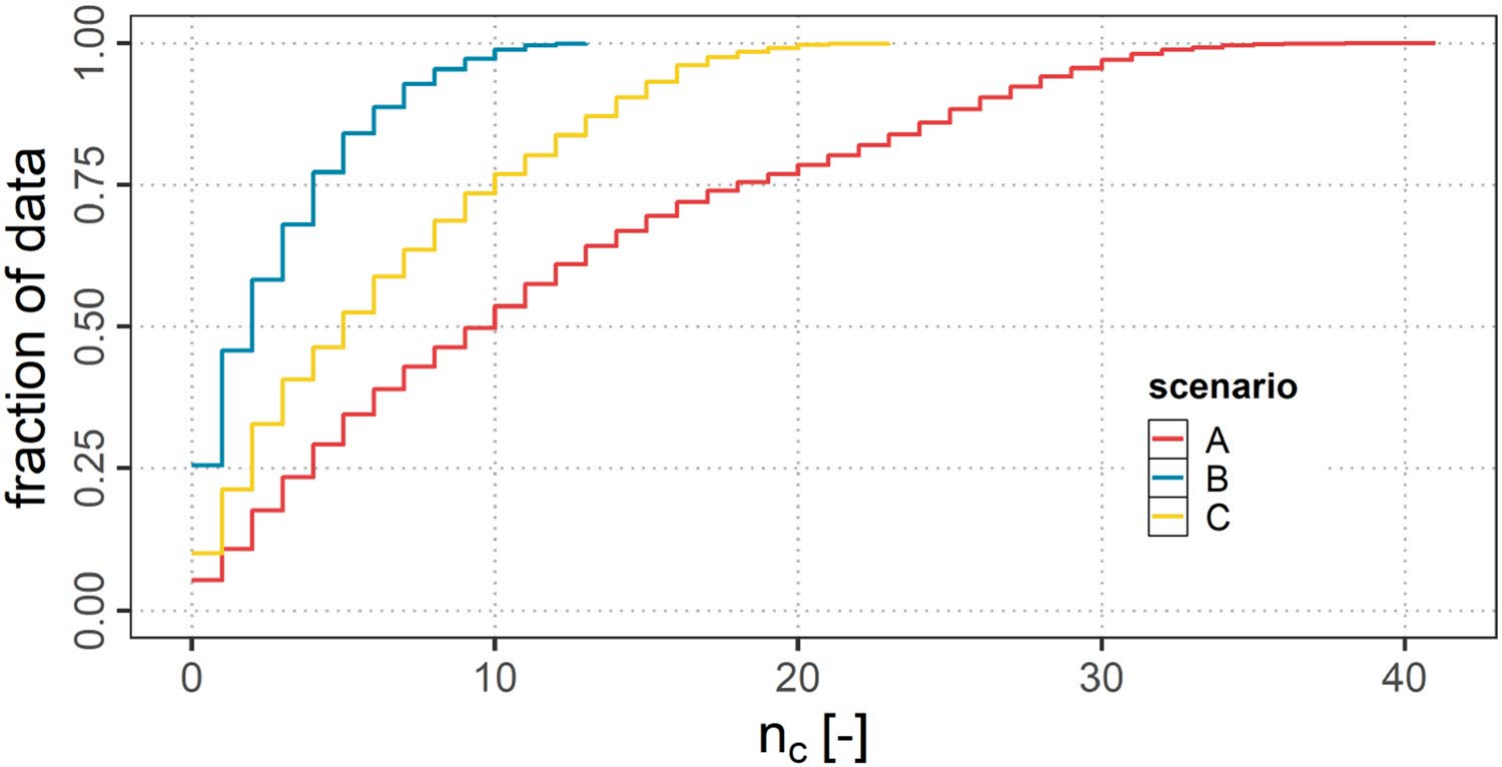
CDF of $n_{c}$ for scenarios A, B and C

Figure 12 shows a CDF of the number of available air connections $n_{c}$ for all aircraft within the ASA. It can be observed, that in scenario A due to the higher communication range $r_{a}$ considerable more aircraft ($n_{c \textrm{max}}=41$) are connected to each other than in scenario B ($n_{c \textrm{max}}=13$) and C ($n_{c \textrm{max}}=23$). Also the amount of aircraft without any air connection is considerably smaller in A and C than in scenario B.

#### Link duration

**Fig. 13:**
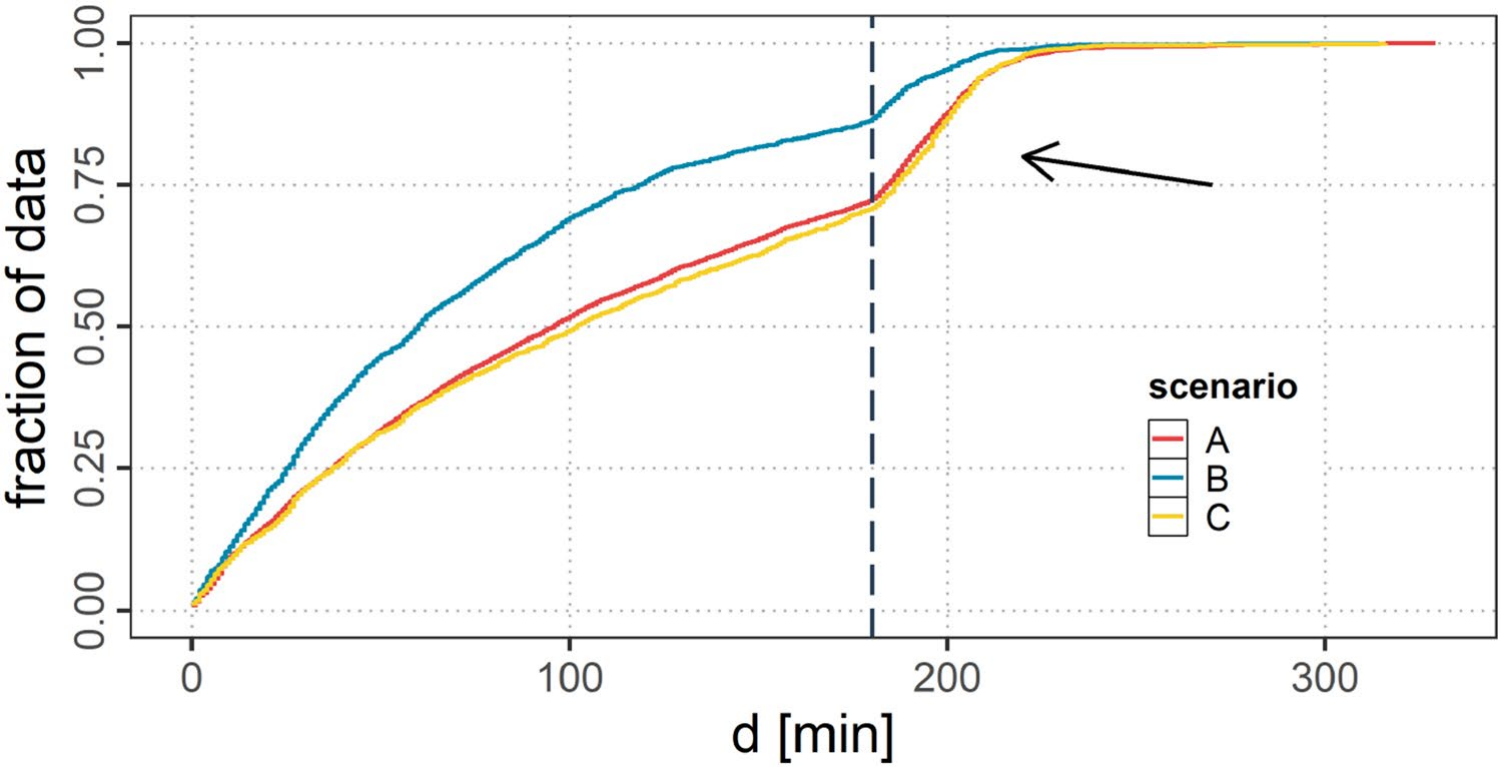
CDF of *d* for scenarios A, B and C; note: direct A2A connections only

Figure 13 shows the CDF of the link durations for all three scenarios. It can be observed that the link durations are not normally distributed. A peak in the longer link durations can be observed (indicated by the vertical line). This is caused by the scenario setup that promotes long link durations as the aircraft fly in the westbound traffic flow almost parallel over the NAT. For scenario B this peak is still observable but not as evident as for scenario A and C as with decreasing $r_{a}$ considerably less partners for the establishment of links can be reached. The average link duration, therefore, is lower in scenario A and C. Scenarios A and C show very similar curves.

### Fraction and range analysis

The findings in the previous section indicate, that ground connectivity and air connectivity are not affected equivalently by variations in $r_{a}$ and $e_\textrm{f}$. Therefore, in this section an aggregated analysis of the connectivity will be presented for different equipage fractions $e_\textrm{f}$ as well as for different maximum A2A communication ranges $r_{a}$ as defined in Table 2. Additionally, the exemplary scenarios as defined in Sect. 4.1 are marked within all presented plots.

#### Ground connectivity

**Fig. 14:**
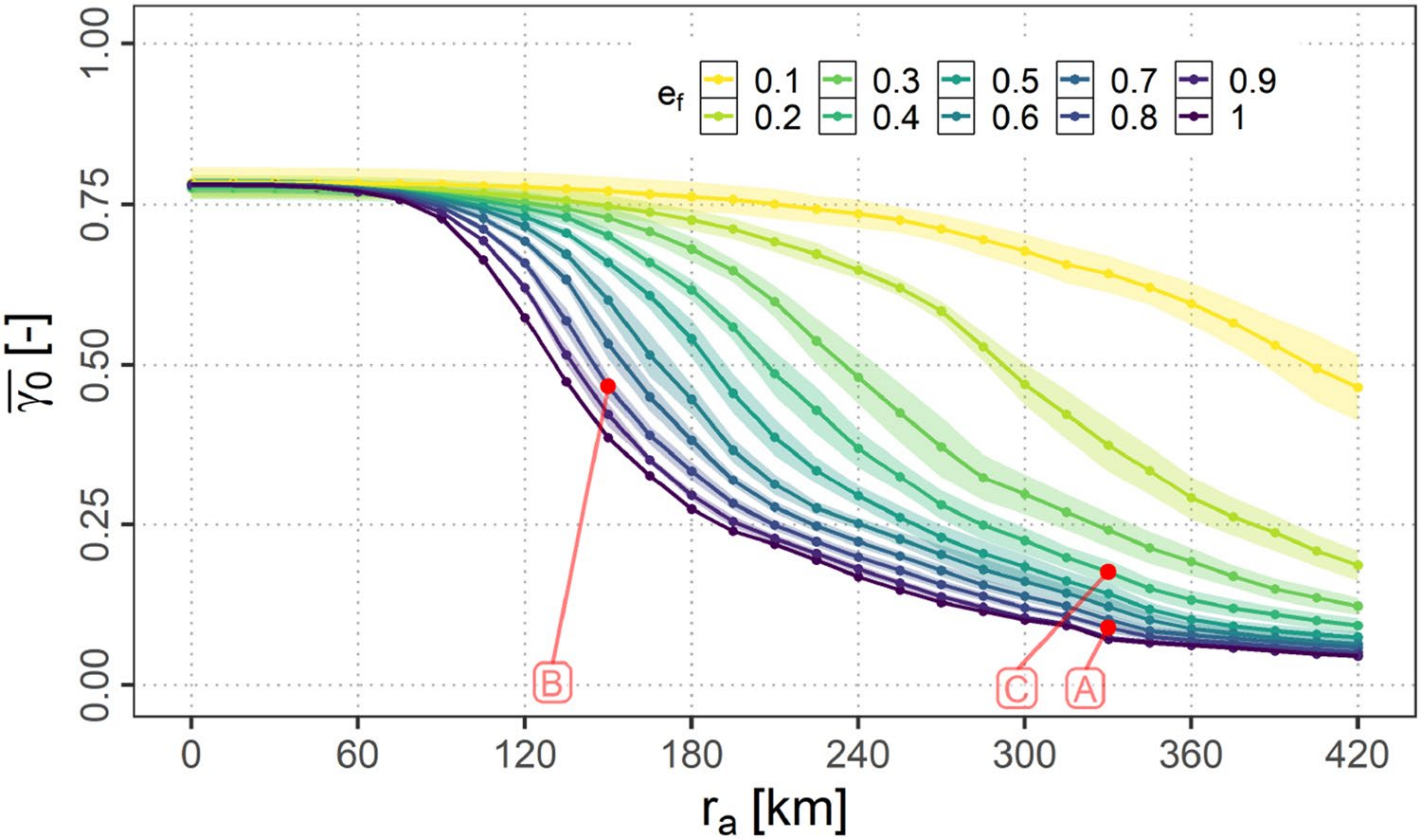
${\bar{\gamma }}_{0}$ for variations of $e_{f}$ and $r_{a}$

Figure 14 shows the means over the samples of the relative ground connectivity per scenario ${\bar{\gamma }}_{0}$ for different equipage fractions $e_\textrm{f}$ and different values for $r_{a}$. The means are marked by a solid line while the 95 % confidence intervals (assuming a normal distribution of the data points) are shown as shaded areas additionally.

It can be observed, that for all fractions the curves start for $r_{a}=0$ at about 0.76 which represents the fraction of airplanes not in direct ground connection. Independent of $e_{f}$ the percentage of flights without ground connection then decreases with an increase of $r_{a}$ after a short period of almost constant values. The higher the fraction of equipped aircraft the earlier the A2G connectivity is established and ${\bar{\gamma }}_{0}$ decreases.

As it can be observed the 95 % confidence intervals for the mean are already very small. As it can be expected this variation decreases with increasing $e_{f}$ as the variability of the samples decrease. For $e_{f}=1$ no variation is possible.

Furthermore, it can be observed from Fig. 14, that the higher the A2A communication range $r_{a}$ the less the equipage fraction $e_{f}$ influences the ground connectivity. Finally ${\bar{\gamma }}_{0}$ can be expected to converge to a threshold for all $e_{f}$ if $r_{a}$ exceeds the radio horizon.

#### Air connectivity

**Fig. 15:**
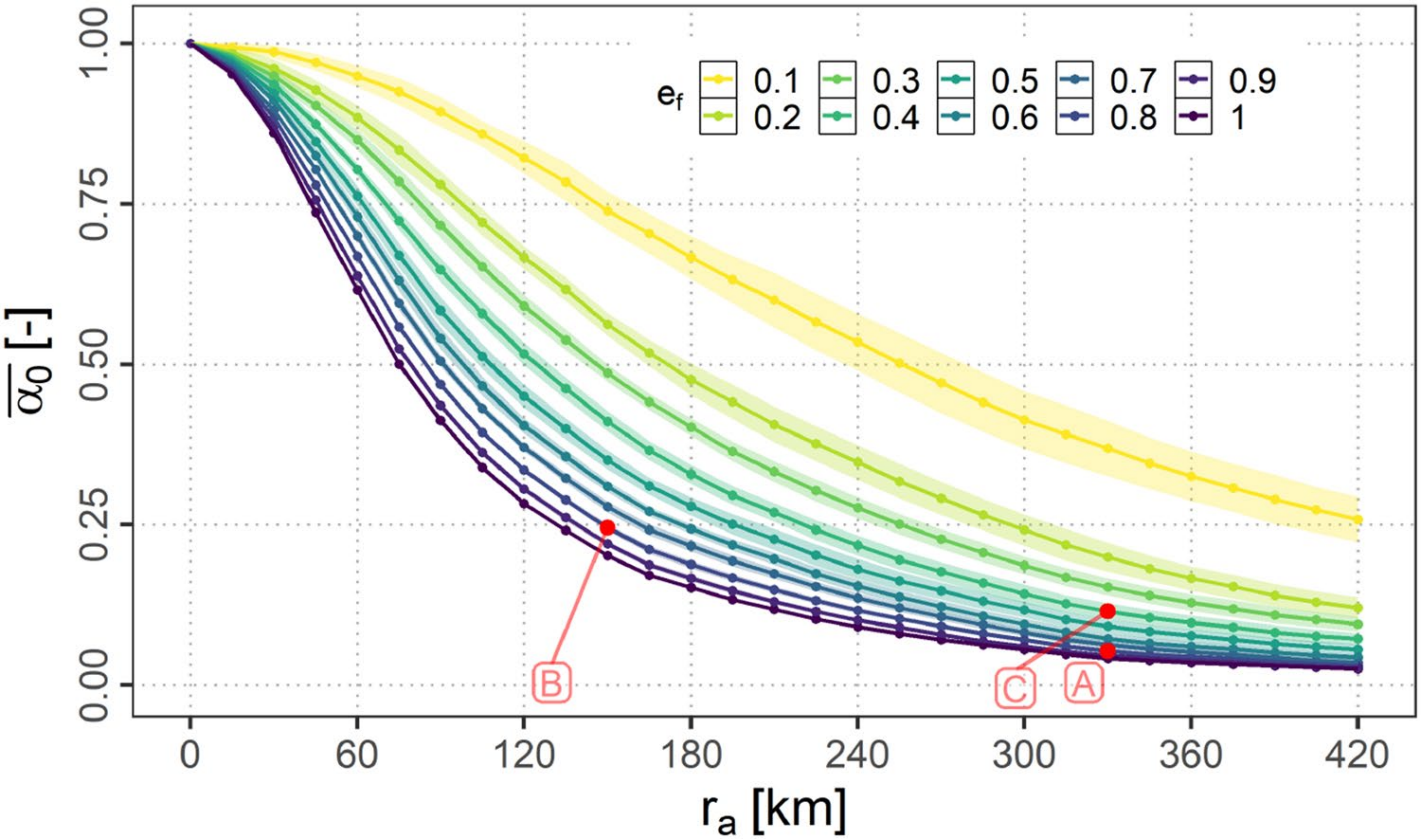
${\bar{\alpha }}_{0}$ for variations of $e_{f}$ and $r_{a}$

Figure 15 shows the means over the samples of the relative air connectivity per scenario ${\bar{\alpha }}_{0}$ for different equipage fractions $e_\textrm{f}$ and A2A communication ranges $r_{a}$. The means are marked by a solid line while the 95 % confidence intervals (assuming a normal distribution of the data points) are shaded.

It can be observed, that independent of $e_\textrm{f}$ the relative air connectivity decreases with an increase of $r_{a}$ beginning at 1 (no air connections at all) for $r_{a}=0$. The higher the fraction of equipped aircraft $e_\textrm{f}$ and the A2A communication range $r_{a}$ the faster connections to other aircraft can be established resulting in a decrease of ${\bar{\alpha }}_{0}$. It can be expected that with increasing $r_{a}$, ${\bar{\alpha }}_{0}$ reaches a threshold for all $e_\textrm{f}$ if $r_{a}$ exceeds the radio horizon.

#### Air and ground connectivity relation

**Fig. 16:**
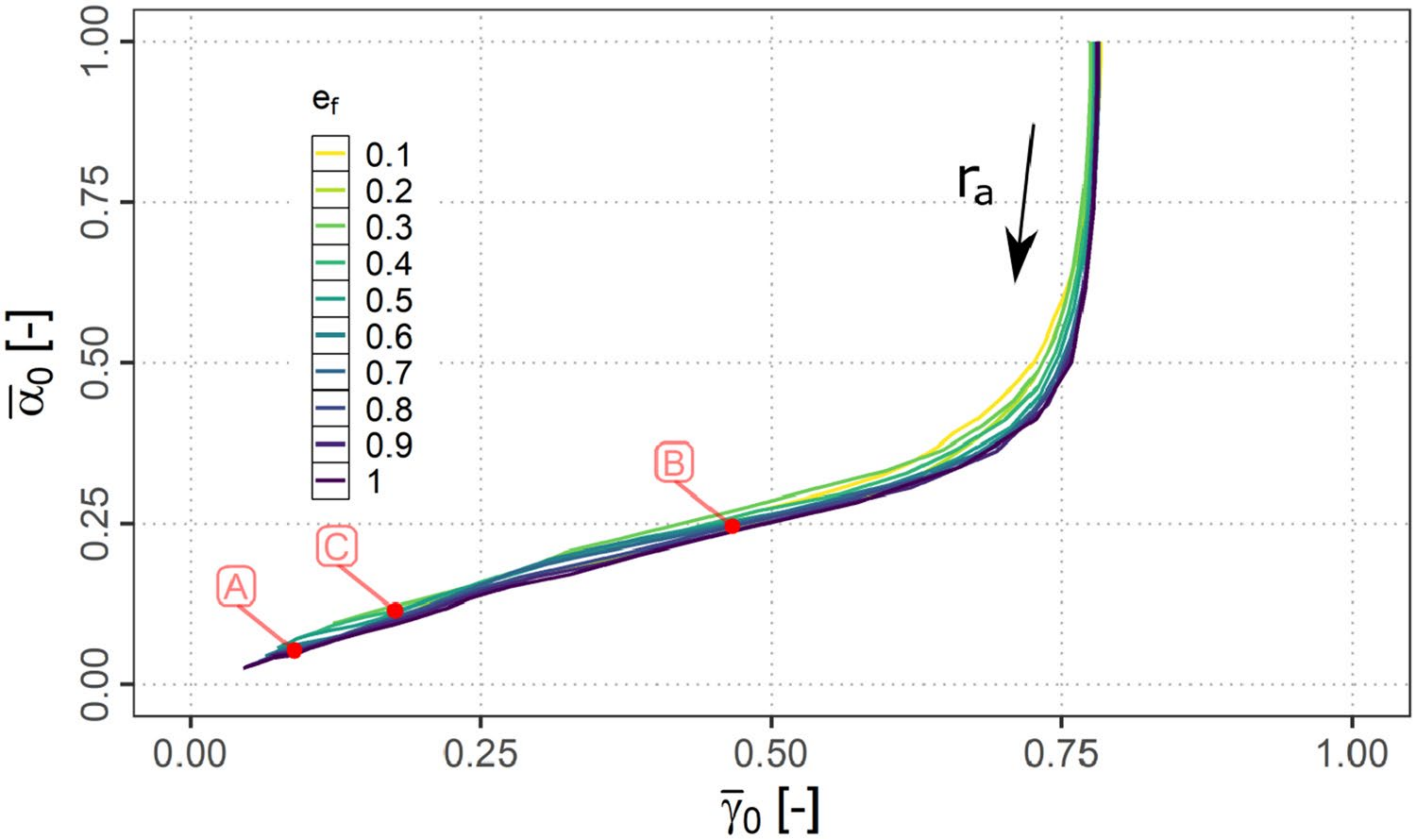
${\bar{\gamma }}_{0}$ vs. ${\bar{\alpha }}_{0}$ for variations of $e_{f}$ and $r_{a}$

It seems reasonable to assume, that the A2A and A2G connectivity are linked to each other as the more air connections can be established, the higher the probability becomes, that a ground connection can be established. Consequently, Fig. 16 shows the relation of ${\bar{\alpha }}_{0}$ and ${\bar{\gamma }}_{0}$ for the fraction means of $e_{f}$ accordingly (note: for better readability confidence intervals are omitted in this plot).

It can be observed, that the curves are comparable for the different $e_{f}$. Beginning with ${\bar{\alpha }}_{0}=1$ (no A2A connections) and a ${\bar{\gamma }}_{0}$ of about 0.76 (all except direct A2G connections) an increase in $r_{a}$ is first only affecting the A2A connectivity. After this first almost linear part a kink in the curves can be observed indicating the onset of the A2G connections being established via the network. After this kink another quasi linear part follows running down towards the origin at which all flights are connected.

#### Hops for A2G connection

**Fig. 17:**
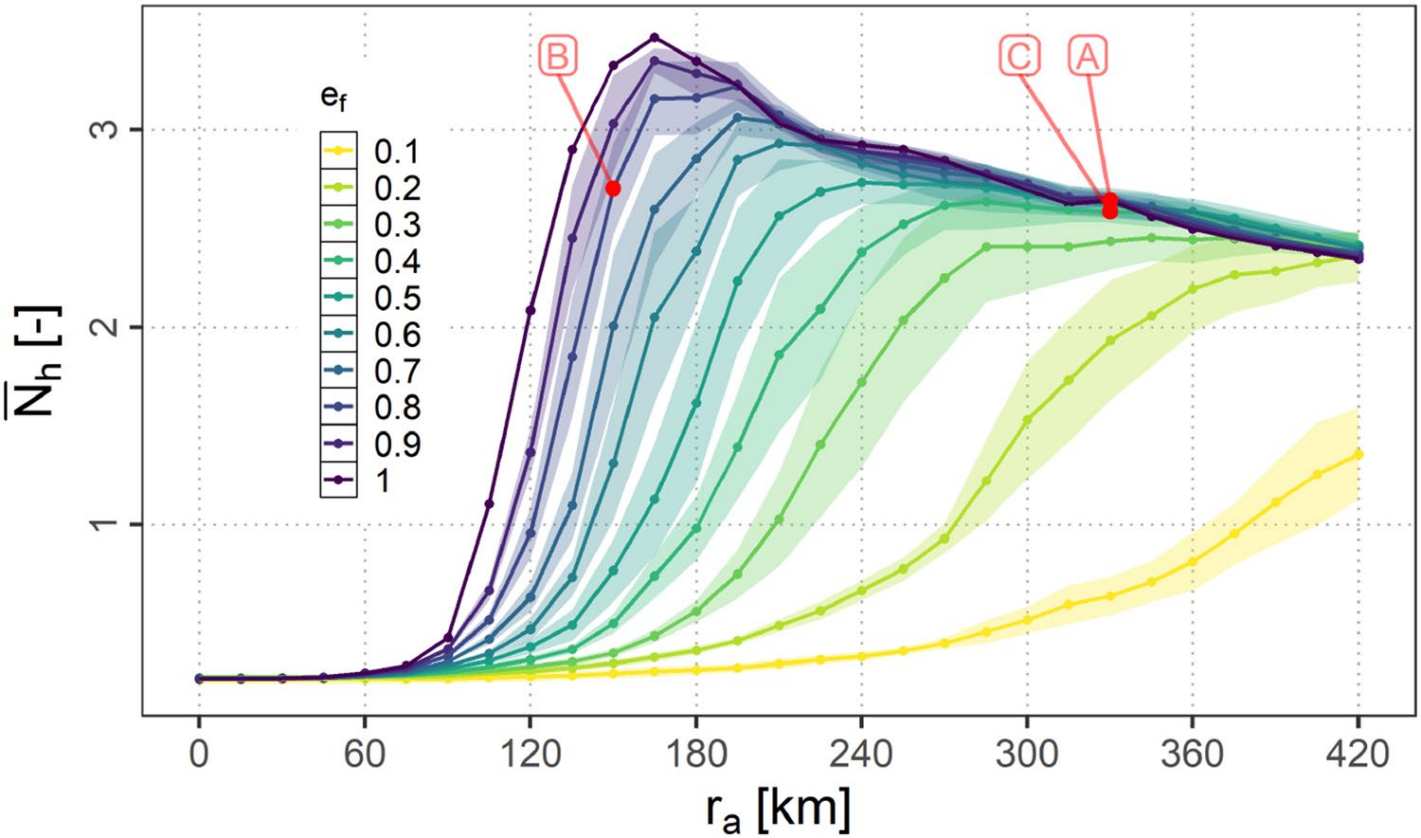
Mean over the samples of the average number of hops per scenario ($\bar{N_{h}}$) for variations of $e_\textrm{f}$ and $r_{a}$

In this section the dependency of the average number of hops $N_{h}$ necessary to establish an A2G connection on $r_{a}$ and $e_{f}$ is presented. Figure 17 shows the mean over the samples of the average number of hops $\bar{N_{h}}$ per scenario for the different equipage fractions along with the 95 % confidence intervals (assuming a normal distribution of the data points) for the means.

It can be observed, that the greater $e_\textrm{f}$ the earlier $\bar{N_{h}}$ rises with $r_{a}$. At a certain value for $r_{a}$ a maximum is reached after that $\bar{N_{h}}$ decreases again. The lower $e_{f}$ the later and the shallower this peak turns out to be. With increasing $r_{a}$ the influence of $e_\textrm{f}$ is diminished.

#### Available connections

**Fig. 18:**
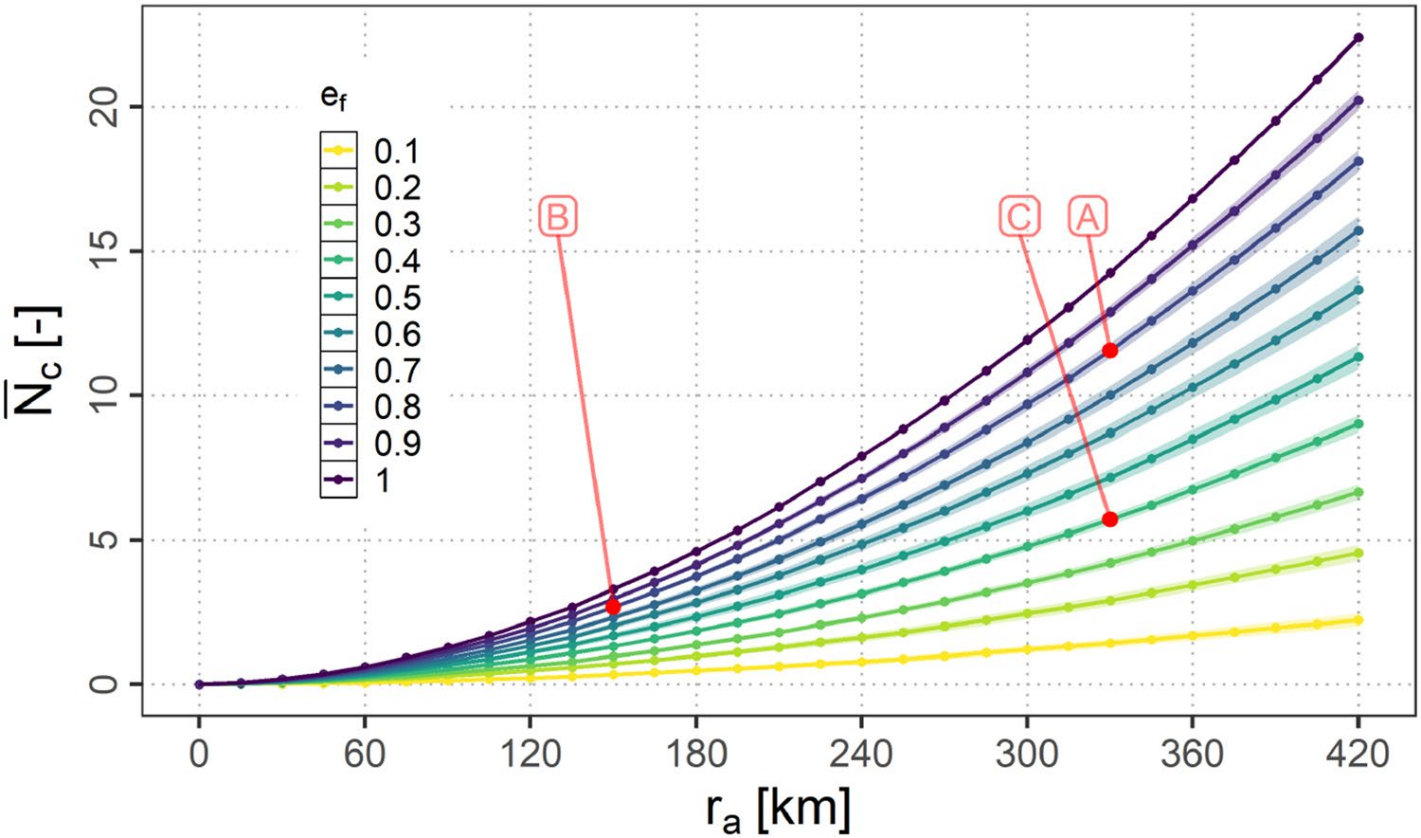
Mean over the samples of the average number of connections per scenario ($\bar{N_{c}}$) for variations of $e_\textrm{f}$ and $r_{a}$

In this section the dependency of the number of available air connections $N_{c}$ on $r_{a}$ and $e_\textrm{f}$ is presented. Figure 18 shows the mean over the samples of the average number of connections $\bar{N_{c}}$ per scenario for the different equipage fractions along with the 95 % confidence intervals (assuming a normal distribution of the data points).

It can be observed, that all curves increase exponentially with $r_{a}$. The lower $e_\textrm{f}$ the less strong this increase turns out to be.

#### Link duration

**Fig. 19:**
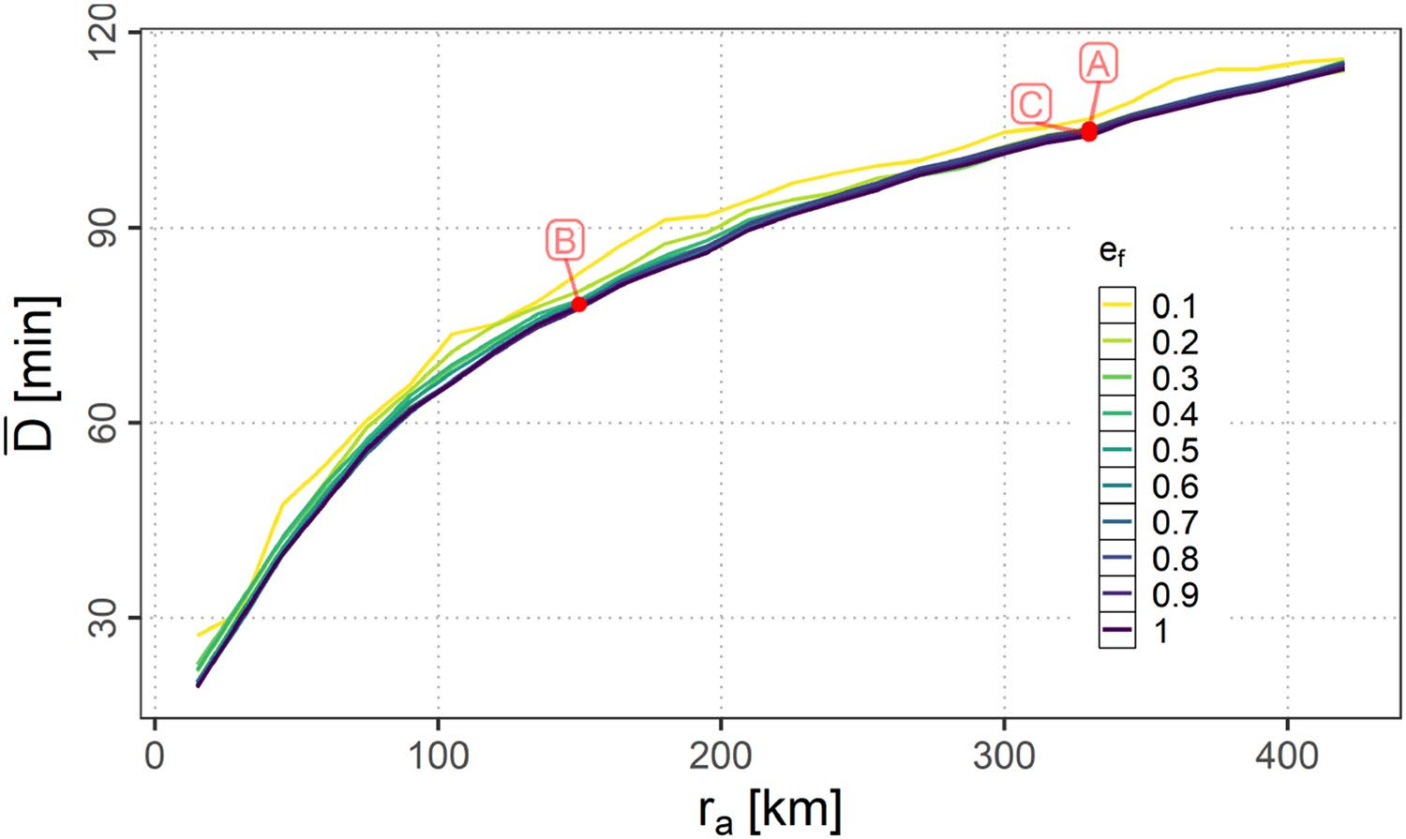
Mean over the samples of the average link durations per scenario (${\bar{D}}$) for variations of $e_\textrm{f}$ and $r_{a}$

In this section the dependency of the link duration *D* on $r_{a}$ and $e_\textrm{f}$ is presented. Figure 19 shows the mean over the samples of the average link duration ${\bar{D}}$ per scenario for the different equipage fractions (note: for reasons of readability the confidence intervals are omitted).

It can be seen, that other than the connectivity metrics, the average link duration changes mainly by $r_{a}$ nearly independently of the equipage fraction $e_\textrm{f}$. This effect can be attributed to the large amount of parallel flights in the modeled scenario as mentioned in Sect. 4.1.5.

### Derivation of requirements

In the design of ad-hoc communication networks and the necessary data link technology there are three central questions as seen from a scenario point of view.

1. Which combinations of equipage fraction and A2A communication range are feasible in order to achieve a required ground connectivity?
2. How large needs the A2A communication range to be in order to achieve a required ground connectivity if a certain amount of aircraft can be assumed to be equipped with the systems?
3. How many aircraft need to be equipped in order to achieve a required ground connectivity if the data link system exhibits a fixed A2A communication range?

**Fig. 20:**
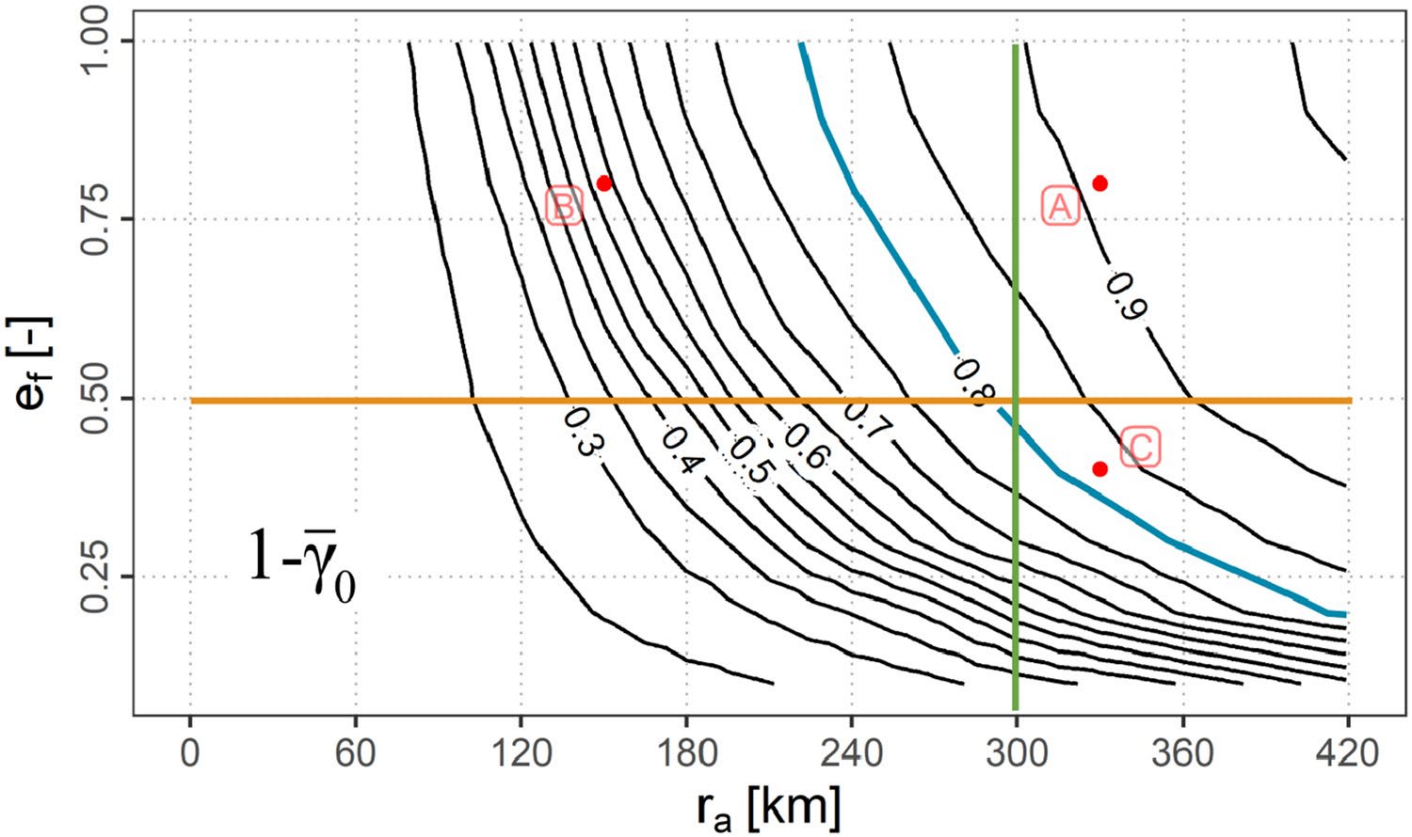
$1 - {\bar{\gamma }}_{0}$ contours depending on $e_\textrm{f}$ and $r_{a}$

The contour plot of $1 - {\bar{\gamma }}_{0}$ as shown in Fig. 20 can be used to answer these questions in a first guess.

As an example all combinations of $e_\textrm{f}$ and $r_{a}$ resulting in 80 % ground connectivity (blue), possible $r_{a}$ and ground connectivities for a fixed $e_\textrm{f}=0.5$ (orange) as well as possible $e_\textrm{f}$ and ground connectivities for a fixed $r_{a}=300\,\textrm{km}$ (green) are marked.

The plot shows, that for lower values of $r_{a}$ (roughly $r_{a}<240\,\textrm{km}$) an increase of $e_\textrm{f}$ is not resulting in a considerably higher ground connectivity, which is the case for higher values of $r_{a}$.

A ground connectivity of 80 % or higher can in turn only be achieved if the communication range exceeds the threshold of about $r_{a}=225\,\textrm{km}$.

**Fig. 21:**
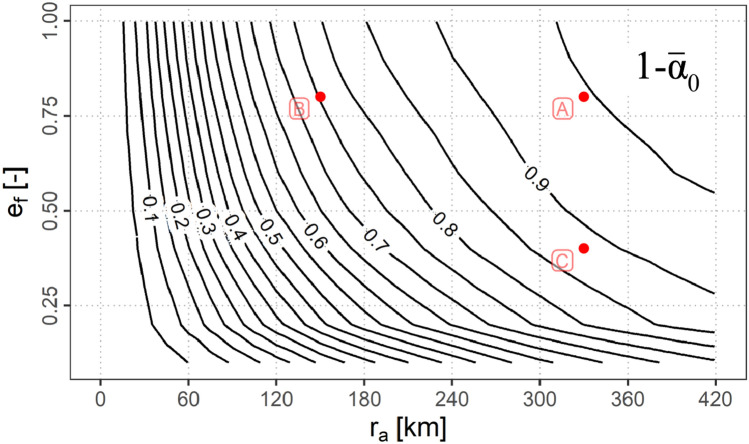
$1-{\bar{\alpha }}_{0}$ contours depending on $e_{f}$ and $r_{a}$

**Fig. 22:**
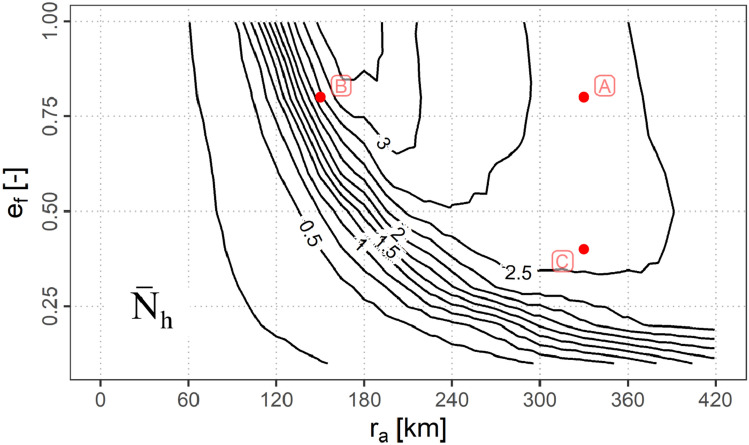
$\bar{N_{h}}$ contours depending on $e_{f}$ and $r_{a}$

**Fig. 23:**
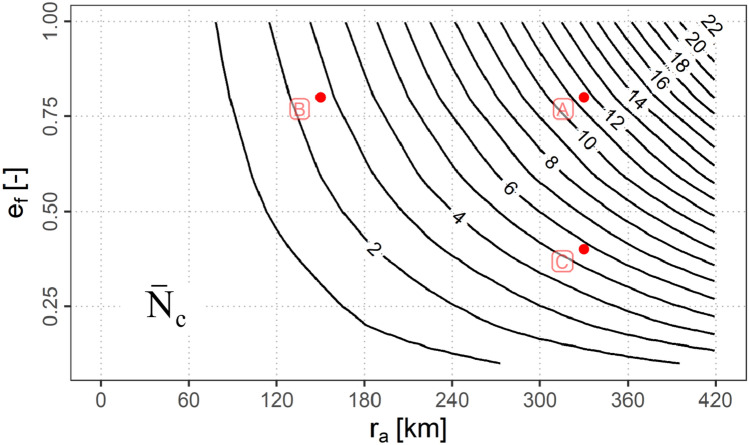
$\bar{N_{c}}$ contours depending on $e_{f}$ and $r_{a}$

In order to derive additional requirements for the data link systems additional contour plots are provided in appendix A. These are the contour plot of $1 - {\bar{\alpha }}_{0}$ (Fig. 21), the contour plot of $\bar{N_{h}}$ (Fig. 22) as well as the contour plot of $\bar{N_{c}}$ (Fig. 23).

## Discussion and outlook

In this paper we presented a modeling approach to model air traffic in the North Atlantic region that can be used for a generic connectivity assessment and for the derivation of geographically triggered data traffic. Additionally, we defined metrics to assess connectivity on scenario level.

For exemplary scenarios observed that that depending on the equipage fraction and the A2A communication range, on the one hand communication bridges can form, that span the whole North Atlantic while on the other hand isolated clusters of aircraft occur without ground connections. Link durations are strongly driven by the parallel character of the flight tracks crossing the North Atlantic.

In a subsequent study we showed, that the equipage fraction and the A2A communication range both have a strong influence on connectivity in terms of total connected flight time, average available connections and average number of hops to reach ground. While assessing average link durations we showed, that for the assessed scenarios the equipage fraction shows a considerable smaller influence on the average link durations in contrast to the A2A communication range that strongly influence this metric.

In addition we showed that A2A or A2G connectivity are not independent from each other and that this relation is strongly depending on the A2A communication range while the equipage fraction only has a minor influence.

In general, our findings enable the statistical derivation of the required equipage fraction and the required communication performance in terms of A2A communication range, average number of hops and average number of connections for a demanded level of A2A or A2G connectivity. This can facilitate the initial design of new communication systems and the specification of technical requirements.

In this respect the data presented in our work indicates, that a required full (100 %) ground connectivity of all aircraft throughout the flight can only be reached if the A2A communication range exceeds 420 km while the equipage fraction needs to reach nearly 100 %. This in fact seems not feasible for future airborne ad-hoc networks that will exhibit considerable lower equipage fractions especially during the initial deployment of the system. However, when relaxing this hard constraint, a ground connectivity requirement of 90 % can in turn lead to A2A communication ranges and equipage fractions that are within the realm of possibility as shown exemplarily in scenario A.

In combination with a data traffic simulator that is developed within the IntAirNet project [26] our model enables the derivation of geographically triggered data messages and the analysis of total data traffic demand for complete scenarios under variation of the parameters.

To further assess connectivity on scenario level as well as to perform an analysis of communication demand coverage by an ad-hoc aeronautical network, it is necessary to perform a detailed analysis of the cluster structure of the resulting connectivity network including the identification of gateways and bottlenecks to estimate the maximum data traffic at the affected connections. This can further help identifying additional requirements for the data link systems under consideration.

As we up to now modeled the westbound traffic flow over the North Atlantic by geodesics, an expansion of our model in terms of adapting the flight routes to follow the organized track system (OTS) will be helpful in the future to asses the whole traffic volume (including the eastbound flights) as well as effects due to the merging of flights on the OTS tracks.

Additionally, a similar study as presented in this paper for the eastbound traffic flow seems reasonable and is deemed to be compared with the findings for the westbound study.

## Data Availability

All data that are not subject to licensing agreements by third parties can be made available by the corresponding author upon reasonable request.

## Apendix 1: Additional contour plots for the derivation of requirements

See Figs. 21, 22 and 23.

## List of symbols

$\alpha$: Relative air connectivity
$\gamma$: Relative ground connectivity
$\Gamma$: Accumulated flight time with ground con. [min]
$\tau$: Overall accumulated flight time [min]
$\varphi$: Amount of aircraft
*a*: Altitude [m]
*A*: Accumulated flight time with air con. [min]
*C*: Connectivity
*d*: Link duration [min]
*D*: Average link durations per scenario
${\bar{D}}$: Mean over samples of average link duration per scenario
$$e_\textrm{f}$$: Equipage fraction
*l*: Link
*L*: Set of all links in scenario
*n*: Amount
*N*: Average number per scenario
${\bar{N}}$: Mean over samples of average number per scenario
*r*: Range [km]
$$R_\textrm{e}$$: Earth radius [m]
*S*: Set of aircraft
*t*: Simulation time [min]

## Indices

*g*: Ground/air-to-ground
*a*: Air/air-to-air
*b*: Boundary
*h*: Hops
*c*: Connections
*LOS*: Line-of-sight
*smp*: Samples

## Abbreviations

A2A: Air-to-air
A2G: Air-to-ground
AAC: Airline Administrative Control
AANET: Aeronautical Ad-hoc Network
AOC: Aeronautical Operational Control
APC: Aeronautical Passenger Communication
AS: Airborne Station
ASA: Applicable Simulation Area
ATC: Air Traffic Control
ATS: Air Traffic Services
CDF: Cumulative Distribution Function
CPDLC: Controller-Pilot Data Link Communication
CSI: Connectivity Simulator
DTG: Data Traffic Generator
FANET: Flying Ad-hoc Network
G2G: Ground-to-ground
GS: Ground Station
HF: High Frequency
ICAO: International Civil Aviation Organization
LDACS: L-band Digital Aeronautical Communications System
LEO: Low Earth Orbit
LOS: Line-of-sight
MANET: Mobile Ad-hoc Network
NAT: North Atlantic
OCA: Oceanic Control Area
ORP: Oceanic, Remote, Polar
OTS: Organized Track System
SATCOM: Satellite Communication
SATS: Small Aircraft Transportation Systems
TCM: Trajectory Calculation Module
UAS: Unmanned Aerial System
VHF: Very High Frequency
VANET: Vehicular Ad-hoc Network

## Declarations

### Code availability

The software code is proprietary information of the German Aerospace Center (DLR). Therefore, the code cannot be made available to the public or the readers without any restrictions. The codes used for analyzing the data and plotting the analyzed data are available from the corresponding author upon reasonable request.
